# Genome-wide bidirectional CRISPR screens identify mucins as host factors modulating SARS-CoV-2 infection

**DOI:** 10.1038/s41588-022-01131-x

**Published:** 2022-07-25

**Authors:** Scott B. Biering, Sylvia A. Sarnik, Eleanor Wang, James R. Zengel, Sarah R. Leist, Alexandra Schäfer, Varun Sathyan, Padraig Hawkins, Kenichi Okuda, Cyrus Tau, Aditya R. Jangid, Connor V. Duffy, Jin Wei, Rodney C. Gilmore, Mia Madel Alfajaro, Madison S. Strine, Xammy Huu Wrynla, Erik Van Dis, Carmelle Catamura, Livia H. Yamashiro, Julia A. Belk, Adam Begeman, Jessica C. Stark, D. Judy Shon, Douglas M. Fox, Shahrzad Ezzatpour, Emily Huang, Nico Olegario, Arjun Rustagi, Allison S. Volmer, Alessandra Livraghi-Butrico, Eddie Wehri, Richard R. Behringer, Dong-Joo Cheon, Julia Schaletzky, Hector C. Aguilar, Andreas S. Puschnik, Brian Button, Benjamin A. Pinsky, Catherine A. Blish, Ralph S. Baric, Wanda K. O’Neal, Carolyn R. Bertozzi, Craig B. Wilen, Richard C. Boucher, Jan E. Carette, Sarah A. Stanley, Eva Harris, Silvana Konermann, Patrick D. Hsu

**Affiliations:** 1 Division of Infectious Diseases and Vaccinology, School of Public Health, University of California, Berkeley, Berkeley, CA USA; 2 Department of Bioengineering, University of California, Berkeley, Berkeley, CA USA; 3 Innovative Genomics Institute, University of California, Berkeley, Berkeley, CA USA; 4 Department of Microbiology and Immunology, Stanford University School of Medicine, Stanford, CA USA; 5 Department of Epidemiology, University of North Carolina at Chapel Hill, Chapel Hill, NC USA; 6 Marsico Lung Institute and Cystic Fibrosis Research Center, University of North Carolina at Chapel Hill, Chapel Hill, NC USA; 7 Department of Biochemistry, Stanford University School of Medicine, Stanford, CA USA; 8 Department of Laboratory Medicine, Yale School of Medicine, New Haven, CT USA; 9 Department of Immunobiology, Yale School of Medicine, New Haven, CT USA; 10 Department of Molecular and Cell Biology, University of California, Berkeley, Berkeley, CA USA; 11 Center for Computational Biology, University of California, Berkeley, Berkeley, CA USA; 12 Department of Chemistry and Stanford ChEM-H, Stanford University, Stanford, CA USA; 13 Department of Microbiology and Immunology, College of Veterinary Medicine, Cornell University, Ithaca, NY USA; 14 Department of Biochemistry and Biophysics, University of North Carolina at Chapel Hill, Chapel Hill, NC USA; 15 Department of Medicine, Division of Infectious Diseases and Geographic Medicine, Stanford University School of Medicine, Stanford, CA USA; 16 The Henry Wheeler Center for Emerging and Neglected Diseases, Berkeley, CA USA; 17 Department of Genetics, University of Texas MD Anderson Cancer Center, Houston, TX USA; 18 Department of Regenerative and Cancer Cell Biology, Albany Medical College, Albany, NY USA; 19 Chan Zuckerberg Biohub, San Francisco, CA USA; 20 Department of Pathology, Stanford University School of Medicine, Stanford, CA USA; 21 Howard Hughes Medical Institute, Stanford University, Stanford, CA USA; 22 Yale Cancer Center, Yale School of Medicine, New Haven, CT USA; 23 Arc Institute, Palo Alto, CA USA

**Keywords:** SARS-CoV-2, Functional genomics, Virology

## Abstract

Severe acute respiratory syndrome coronavirus 2 (SARS-CoV-2) causes a range of symptoms in infected individuals, from mild respiratory illness to acute respiratory distress syndrome. A systematic understanding of host factors influencing viral infection is critical to elucidate SARS-CoV-2–host interactions and the progression of Coronavirus disease 2019 (COVID-19). Here, we conducted genome-wide CRISPR knockout and activation screens in human lung epithelial cells with endogenous expression of the SARS-CoV-2 entry factors *ACE2* and *TMPRSS2*. We uncovered proviral and antiviral factors across highly interconnected host pathways, including clathrin transport, inflammatory signaling, cell-cycle regulation, and transcriptional and epigenetic regulation. We further identified mucins, a family of high molecular weight glycoproteins, as a prominent viral restriction network that inhibits SARS-CoV-2 infection in vitro and in murine models. These mucins also inhibit infection of diverse respiratory viruses. This functional landscape of SARS-CoV-2 host factors provides a physiologically relevant starting point for new host-directed therapeutics and highlights airway mucins as a host defense mechanism.

## Abstract (web summary)

Genome-wide CRISPR knockout and activation screens in human lung epithelial cells with endogenous expression of the SARS-CoV-2 entry factors *ACE2* and *TMPRSS2* identify mucins as key host factors restricting viral infection.

## Main

SARS-CoV-2 is a positive-sense RNA Betacoronavirus, belonging to the Coronaviridae family along with other human respiratory pathogens, including the causative agents of severe acute respiratory syndrome (SARS) and Middle East respiratory syndrome (MERS)^1–3^. COVID-19, caused by SARS-CoV-2, can manifest with a diverse set of clinical outcomes ranging from fever and flu-like symptoms in nonsevere cases to acute lung injury and acute respiratory distress syndrome in severe cases^4,5^.

SARS-CoV-2 infects cells by binding to the angiotensin-converting enzyme II (ACE2) receptor. The SARS-CoV-2 Spike (S) glycoprotein is primed to initiate virus–cell fusion through a proteolytic cleavage event, which can be mediated by transmembrane serine protease 2 (TMPRSS2) on the cell surface, or in its absence by the lysosomal endopeptidase cathepsin L (CTSL) following clathrin-mediated endocytosis^6–8^. The TMPRSS2-mediated cell-surface entry route is considered dominant for SARS-CoV-2 in lung epithelial cells because inhibition of TMPRSS2, but not CTSL, in primary lung epithelial cells is sufficient to inhibit viral infection^9^. Further, *ACE2* and *TMPRSS2* are largely co-expressed by the main cellular targets of SARS-CoV-2 in vivo, such as epithelial cells within the lower and upper airway, the nasal passage and the gut^10,11^.

Each step of the viral life cycle is influenced, either positively or negatively, by a vast array of host factors. Although recent loss-of-function (LOF) screens have begun defining host factor requirements for SARS-CoV-2 infection, these studies employed host gene knockout (KO) approaches either in nonepithelial cell lines or in cell lines that do not endogenously express *ACE2* and *TMPRSS2* (refs. ^8,12–16^). Although LOF screens can be powerful for the identification of proviral genes, gain-of-function (GOF) screens can identify antiviral factors that mediate viral restriction upon upregulation. Performing screens in a bidirectional manner can therefore illuminate host pathways with bimodal roles and provide a more comprehensive view of viral dependencies and potential targets for host-directed therapeutic development. We therefore set out to define the host–pathogen interactions required for facilitating or restricting SARS-CoV-2 infection in Calu-3 cells, a human lung cell line endogenously expressing both *ACE2* and *TMPRSS2*.

We conducted genome-scale LOF and GOF CRISPR screens to generate a systematic functional map of host dependencies and host restriction factors. Pathway analysis and secondary validation of top screen hits revealed diverse cellular components involved in modulating cellular proliferation, intercellular junctional complexes, the cytoskeleton, inflammatory signaling and mucin glycoproteins. The gene hits identified in our bidirectional dataset are differentially expressed in single-cell RNA sequencing (scRNA-seq) datasets of lung epithelial cells from healthy individuals and COVID-19 patients, underscoring their physiological relevance.

We further demonstrate an antiviral role for membrane-anchored mucins in vitro and in mouse models of SARS-CoV-2 infection, and find that this antiviral activity extends to diverse respiratory viruses. Taken together, our bidirectional CRISPR screens dissect the densely interconnected landscape of proviral and antiviral host factors, highlighting mucins as potential restriction factors of SARS-CoV-2 infection.

## Identification of proviral and antiviral SARS-CoV-2 host factors

**Fig. 1:**
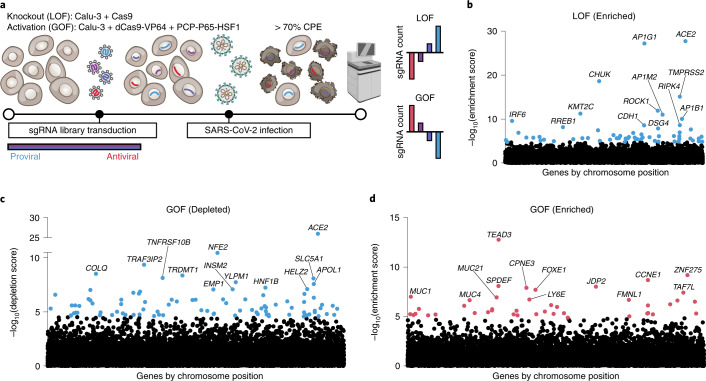
Bidirectional CRISPR screens identify host factors critical for SARS-CoV-2-mediated CPE. **a**, Schematic of genome-wide CRISPR KO and activation screens for SARS-CoV-2 host factors, conducted in parallel. Calu-3 cells stably expressing Cas9 for the LOF screen or dCas9 and transcriptional activators for the GOF screen were transduced with pooled guide RNA libraries. Following infection with SARS-CoV-2, cells were harvested after at least 70% CPE was evident. Next-generation sequencing was performed to identify host factors and assign proviral and antiviral roles based on guide RNA enrichment or depletion compared with uninfected controls. **b**, Manhattan plot displaying the top 13 enriched genes identified in the LOF screen. **c**, Manhattan plot displaying the top 13 depleted genes in the GOF screen. **d**, Manhattan plot displaying the top 13 enriched genes in the GOF screen. All genes are ranked based on MAGeCK robust rank aggregation (RRA) score. Red dots indicate putative antiviral genes with FDR < 0.05, blue dots indicate putative proviral genes with FDR < 0.05.

**Extended Data Fig. 1:**
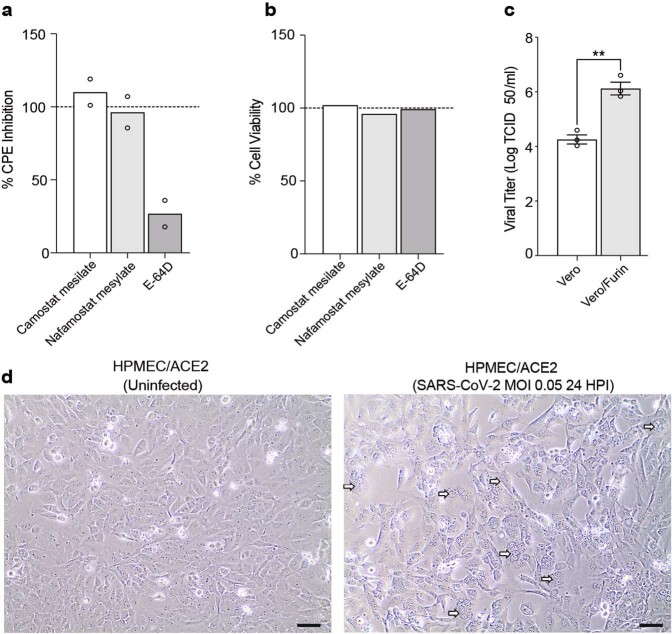
Characterization of SARS-CoV-2 virus stocks. **a**. Cytopathic effect inhibition assay measuring the capacity of 40 µM of the indicated compound to inhibit SARS-CoV-2-mediated cell death. Calu-3 cells were infected with SARS-CoV-2 at an MOI of 0.05, and cell death was measured 96 hours post-infection. Significance was not calculated, as n = 2 biological replicates. **b**. Levels of compound-induced cytotoxicity in the absence of SARS-CoV-2. **c**. Viral infection of parental Vero-E6 cells or Vero-E6 cells overexpressing furin. Cells were infected with SARS-CoV-2 at an MOI of 0.05, and infectious viral particles were quantified by TCID_50_ 24 hours post infection (hpi). Significance was determined by two-sided t-test, n = 3 biological replicates. **d**. Human pulmonary microvascular endothelial cells (HPMEC) overexpressing human ACE2 (HPMEC/ACE2) were infected with SARS-CoV-2 at an MOI of 0.05, and bright field microscopy images were captured at 10X magnification. Scale bar indicates 500 micrometers. Arrows indicate multinucleated syncytia. These images are representative of n = 3 biological replicates. **For all panels**: error bars denote mean ± s.e.m., significance is indicated as: n.s.=not significant, *= P < 0.05, **= P < 0.01, ***=P < 0.001, ****= P < 0.0001.

To systematically define proviral and antiviral host factors in a physiologically relevant cellular context, we conducted both LOF and GOF CRISPR screens using virus-mediated cell death as a functional readout (Fig. 1)^17^. We utilized a human lung epithelial cell line (Calu-3) that expresses *ACE2* and *TMPRSS2*, is highly permissive to SARS-CoV-2 infection, and exhibits a cytopathic effect (CPE). We first transduced Calu-3 cells with constructs encoding Cas9 nuclease (LOF) or the synergistic activation mediator transcriptional activators dCas9–VP64 + PP7-P65-HSF1 (GOF), followed by lentiviral delivery of CRISPR guide libraries at low multiplicity of infection (MOI) (Fig. 1a)^18,19^. We confirmed that our SARS-CoV-2 isolate possessed a functional furin cleavage site and infected cells in a manner dependent on TMPRSS2 (and independent of CTSL) in this cell line (Extended Data Fig. 1).

**Extended Data Fig. 2:**
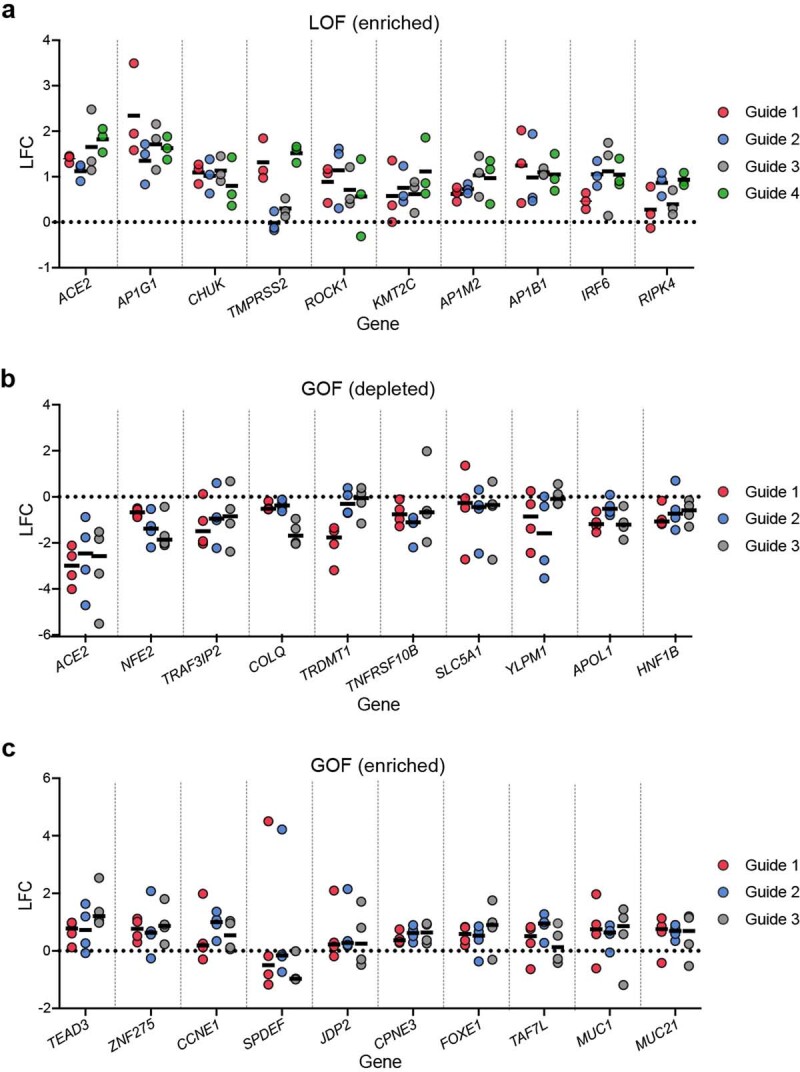
Quality control metrics for bidirectional SARS-CoV-2 host factor screens. **a**. Log fold-change (LFC) of top screen hits enriched in the LOF screen. Guides are shown separately, with screen replicates indicated by individual data points (log fold-change is calculated relative to day 0 pre-infection of the same cell population). n = 3 biological screen replicates. **b**. Log fold-change of top screen hits depleted in the gain-of-function screen. n = 4 biological screen replicates. **c**. Log fold-change of top screen hits enriched in the GOF screen. n = 4 biological screen replicates.

Our LOF and GOF screens were performed with three and four biological replicates, respectively, while maintaining greater than 1,000× single guide RNA (sgRNA) coverage (Extended Data Fig. 2). Following SARS-CoV-2 infection, genomic DNA was harvested after at least 70% CPE was observed (~5 d post infection). Next-generation sequencing of sgRNAs enabled identification of genes enriched or depleted relative to uninfected control cells (Supplementary Tables 1–3). We interpreted genes emerging from the LOF-enriched (Fig. 1b) and GOF-depleted (Fig. 1c) categories to be proviral, and genes from the GOF-enriched category (Fig. 1d) to be antiviral. We omitted further analysis of the LOF-depleted genes because of overlap of gene hits with essential genes, which would confound our analysis.

The top gene from both the LOF-enriched and GOF-depleted screens was *ACE2*, the SARS-CoV-2 cell entry receptor, validating the phenotypic selection of the host factor screens. *TMPRSS2* was also highly enriched in our LOF screen, further confirming that SARS-CoV-2 infection of Calu-3 cells is dependent on the dominant SARS-CoV-2 S glycoprotein-priming mechanism in lung epithelial cells^9,20^. Other top proviral gene hits included *AP1G1* (encoding a clathrin adapter) and *CHUK* (encoding IKK-alpha of the nuclear factor-κB (NF-κB) signaling pathway) in the LOF-enriched analysis, as well as *NFE2* (encoding a transcription factor) and *TRAF3IP*2 (encoding an E3 ubiquitin ligase involved in interleukin 17 and NF-κB signaling pathways) in the GOF-depleted approach (Fig. 1b,c)^21–26^. The NF-κB pathway is commonly activated by viral infections and is known to regulate proinflammatory cytokine production, whereas the clathrin-mediated endocytosis pathway is directly involved in SARS-CoV-2 entry. Interleukin 17 signaling may be directly proviral by establishing a cellular environment conducive to viral replication or may negatively affect the host cell by promoting cell death^27–31^.

**Fig. 2:**
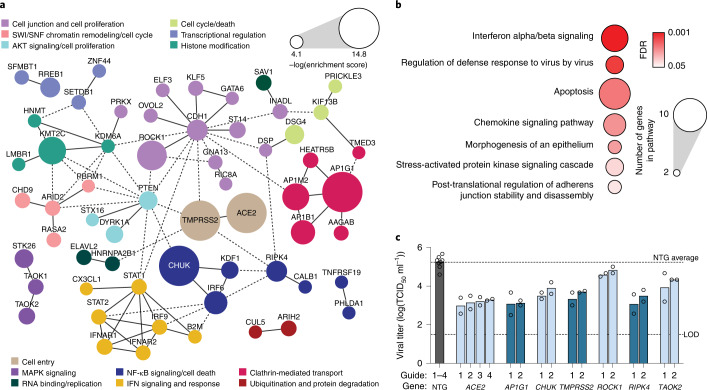
Host dependency factors and pathways of SARS-CoV-2 in lung cells revealed by genome-wide LOF screening. **a**, Protein–protein interaction network for top 100 enriched hits identified in the CRISPR LOF screen based on STRING analysis. Solid lines between genes indicate direct interaction, dashed lines indicate indirect connections. Nodes are color-coded by functional groups and scaled according to screen enrichment RRA score. AKT, AK strain transforming; IFN, interferon; MAPK, mitogen-activated protein kinase; SWI/SNF; SWItch/Sucrose Non-Fermentable. **b**, Pathway analysis of top 100 enriched hits indicates significantly overrepresented pathways with putative proviral roles. Circle size indicates the number of genes within each pathway, color indicates FDR of pathway enrichment. **c**, Individual TCID_50_ validation for KO of the top five enriched hits from our LOF screen plus additional hits of interest. Calu-3 cells were infected with SARS-CoV-2 at an MOI of 0.05 for 48 h, each gene was targeted with at least two separate guides. Dotted lines indicate the limit of detection (LOD) of the assay as well as the NTG average for four separate guides. Significance was not calculated because *n* = 2 biological replicates.

On the antiviral side, our GOF screen identified *TEAD3*, *CCNE1* and *ZNF275* as the top three enriched genes (Fig. 1d and Supplementary Table 3). *TEAD3* encodes a transcription factor involved in the transforming growth factor-β and Hippo signaling pathways, which can regulate cell proliferation^32^. Hippo signaling is activated by diverse stimuli including viral infection and is regulated through kinases such as the LOF-enriched hits *TAOK1* and *TAOK2* (Fig. 2a,c)^33^. *CCNE1* encodes cyclin E1, a regulatory subunit of cyclin-dependent kinase 2, which is required for the G1/S cell-cycle transition^34^. Because numerous studies have reported cell-cycle arrest as a requirement for optimal viral replication, enhanced cell proliferation resulting from *CCNE1* overexpression is likely refractory to SARS-CoV-2 replication^35–37^. In fact, the SARS-CoV-1 N protein has been previously shown to inhibit CCNE1, suggested to be a strategy of viral-mediated cell-cycle arrest to route cellular resources toward viral replication^38^. Finally, *ZNF275* is a zinc-finger protein that may be involved in transcriptional regulation^39^. Taken together, viral entry and trafficking factors, components involved in proinflammatory responses and cell proliferation regulators are critical determinants of SARS-CoV-2-mediated cell death in Calu-3 cells.

## SARS-CoV-2 host dependency pathways and interaction networks

**Extended Data Fig. 3:**
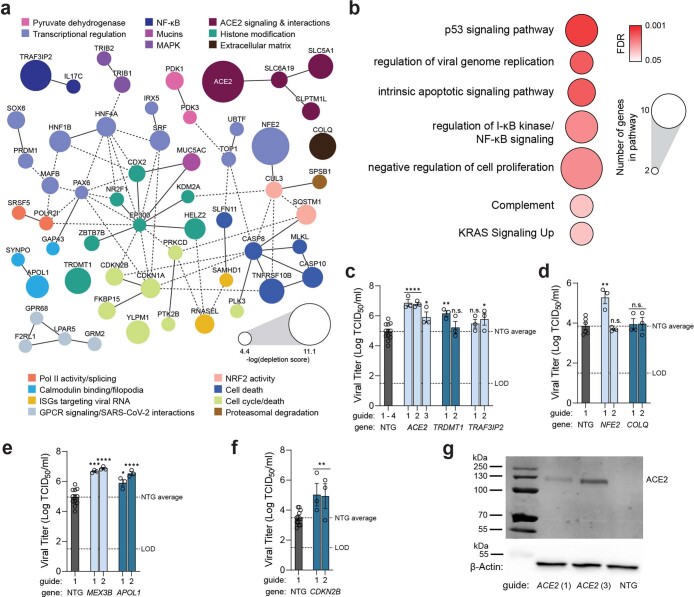
SARS-CoV-2 proviral signatures revealed by depleted genes in the GOF screen. **a**. Protein-protein interaction network for top 100 depleted hits from the CRISPR GOF screen based on STRING analysis. Solid lines between genes indicate direct interaction, dashed lines indicate indirect connections. Nodes are color-coded by functional groups and scaled according to screen enrichment RRA score. **b**. Pathway analysis of top 100 depleted hits in the GOF screen indicates enriched pathways with putative antiviral roles. Circle size indicates the number of genes within each pathway, color indicates FDR of pathway enrichment. **c-d**. Individual guide TCID_50_ validation of the effect of transcriptional upregulation of the top five putative proviral hits on SARS-CoV-2 viral titer in Calu-3 cells infected at an MOI of 0.05 for 48 hours. Each gene was targeted with two sgRNAs. Dotted line indicates the limit of detection (LOD) of the assay and average titer of NTG lines tested. Significance was determined by a two-sided t-test comparing each GOF cell line to the NTG control cells, n = 3 biological replicates. **e-f**. Same as (c-d) but with additional proviral hits of interest. Significance was determined by a two-sided t-test comparing each GOF cell line to the NTG control cells, n = 3. Dotted line indicates the LOD of the assay and average titer of NTG lines. **g**. Western blot analysis of Calu-3 ACE2 GOF cells. One western blot was conducted in this experiment, then re-probed for β-actin as a loading control. **For all panels**: error bars denote mean ± s.e.m., significance is indicated as: n.s.=not significant, *= P < 0.05, **= P < 0.01, ***=P < 0.001, ****= P < 0.0001. Source data

To gain systematic insight into critical proviral host pathways and interactions for SARS-CoV-2 infection, we conducted two analyses of our top 100 enriched and depleted hits from our LOF and GOF screens, respectively. First, we performed a protein–protein interaction network analysis to define putative interactions and group hits into distinct interaction clusters based on both direct and indirect connections (Fig. 2a and Extended Data Fig. 3a). Next, we performed gene set enrichment analysis to identify enriched biological pathways (Fig. 2b and Extended Data Fig. 3b). We identified apoptotic signaling pathways that are expected to be directly involved in mediating virus-induced CPE, as well as canonical interferon signaling that could impact CPE through modulation of cell proliferation or cell death pathways. We also found components of NF-κB-mediated inflammatory signaling, cell–cell junctional complexes, cytoskeletal remodeling, adapter-mediated clathrin transport and cell-cycle regulation as enriched in our proviral screens across highly interconnected networks in Calu-3 cells (Fig. 2a and Extended Data Fig. 3a).

**Extended Data Fig. 4:**
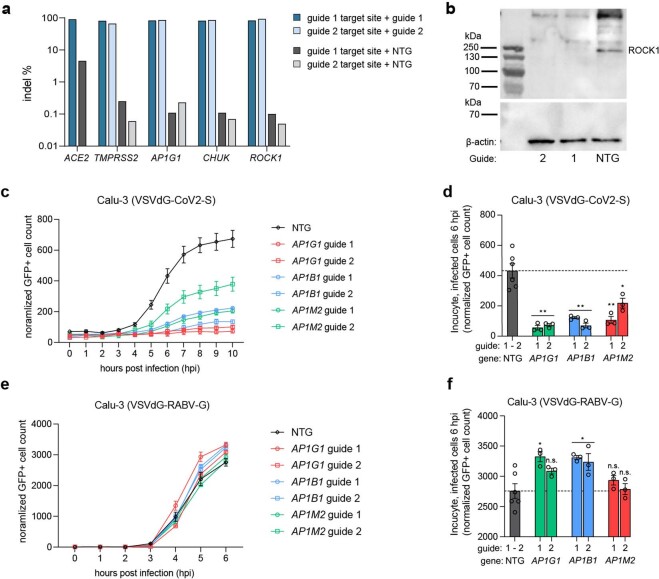
Confirmation of gene editing efficiency for LOF cells and loss of AP1 adaptor complex components decreases Spike glycoprotein-pseudotyped viral entry. **a**. Next generation sequencing was performed to analyze indel percentage in the indicated LOF cell Calu-3 lines. Graph depicts the indel percentage for each guide compared to NTG editing efficiency at the same locus. **b**. Western blot analysis for a representative KO target ROCK1 indicates successful depletion of the target gene for two guides. One western blot was conducted in this experiment, then re-probed for β-actin as a loading control **c**. Time-course of VSV-CoV-2-S infection of NTG, *AP1G1, AP1B1* and *AP1M2* KO cell lines. NTG, non-targeting guide. VSV-CoV-2-S, SARS-CoV-2 Spike-pseudotyped vesicular stomatitis virus encoding GFP. n = 3 biological replicates. **d**. Number of GFP + cells at 6 hpi, from the time-course shown in (c). Statistical significance was measured by individual two-sided t-tests comparing GOF lines to NTG lines, n = 3 biological replicates. **e**. Time-course of VSV-RABV-G entry in NTG, *AP1G1, AP1B1* and *AP1M2* KO cell lines. VSV-RABV-G, VSV particles pseudotyped with rabies virus glycoprotein. n = 3 biological replicates. **f**. Number of GFP + cells at 6 hpi, from the time-course shown in **e**. Statistical significance is measured by individual two-sided t-tests comparing GOF lines to NTG, n = 3 biological replicates. **For all panels**: error bars denote mean ± s.e.m., significance is indicated as: n.s.=not significant, *= P < 0.05, **= P < 0.01, ***=P < 0.001, ****= P < 0.0001. Source data

To confirm the impact of specific genes on SARS-CoV-2 infection, we generated individual Calu-3 KO lines with two distinct guides per gene for the top five LOF-enriched hits and GOF-depleted gene hits—genes predicted to be critical for SARS-CoV-2 infection—and confirmed successful gene editing (Extended Data Fig. 4a,b). Assaying these cell lines using a median tissue culture infectious dose (TCID_50_) assay, we observed reduced infectious virus production when *ACE2, TMPRSS2*, *AP1G1*, *CHUK*, *ROCK1*, *RIPK4* and *TAOK2* genes were disrupted (Fig. 2c). The observed proviral effects of *RIPK4* and *CHUK* suggest that certain components of NF-kB signaling are beneficial for SARS-CoV-2 infection and may be actively regulated by the virus to promote a viral replicative niche. It has been previously observed that NF-kB pathways can have both proviral and antiviral roles, and can be actively regulated and rerouted by other coronaviruses and influenza A^30,40^.

For validation of our top five GOF-depleted hits, lentiviral transduction of CRISPR activation components confirmed that upregulation of *ACE2*, *NFE2*, *TRAF3IP2* and *TRDMT1* increased viral infection to varying levels relative to the nontargeting guide (NTG) control (Extended Data Fig. 3c,d). By contrast, *COLQ* upregulation had no impact on viral replication in our TCID_50_ assay (Extended Data Fig. 3d). Additional GOF-depleted hits including *MEX3B*, *APOL1* and *CDKN2B* also increased viral infection compared with NTG control cells (Extended Data Fig. 3e,f). As expected, western blot analysis confirmed successful overexpression of *ACE2* (Extended Data Fig. 3g). *CDKN2B* encodes for the cyclin-dependent kinase inhibitor 2B, which controls the progression from G1 to S phase. Negative regulation of cell proliferation was the top pathway found among the depleted genes of the GOF screen (Extended Data Fig. 3b), supported by previous observations that the nucleocapsid protein (N) of SARS-CoV-1 actively inhibits cyclin-dependent kinases to arrest the cell cycle^38^.

One of our top hits confirmed by this analysis, *AP1G1*, encodes a subunit of the clathrin adapter complex AP1, and our proviral screens further identified two other subunits of AP1 (AP1M2, AP1B1) as well as known direct interaction partners (*HEATR5B*, *AAGAB*) (Fig. 2a). We hypothesized that AP1 components are required for SARS-CoV-2 cellular entry, given the known role of clathrin-mediated endocytosis as an entry route. To confirm this, we used vesicular stomatitis virus (VSV) encoding green fluorescent protein (GFP) and pseudotyped with SARS-CoV-2 S protein (VSV-CoV-2-S) to infect cells lacking AP1G1, AP1M2 or AP1B1, and observed attenuated viral infection relative to a NTG control (Extended Data Fig. 4c,d). By contrast, these AP1 components were not required for infection of VSV pseudotyped with the rabies virus glycoprotein (VSV-RABV-G), suggesting a specific requirement for AP1 components for SARS-CoV-2 entry (Extended Data Fig. 4e,f).

## Unique SARS-CoV-2 dependencies in lung epithelial cells

**Fig. 3:**
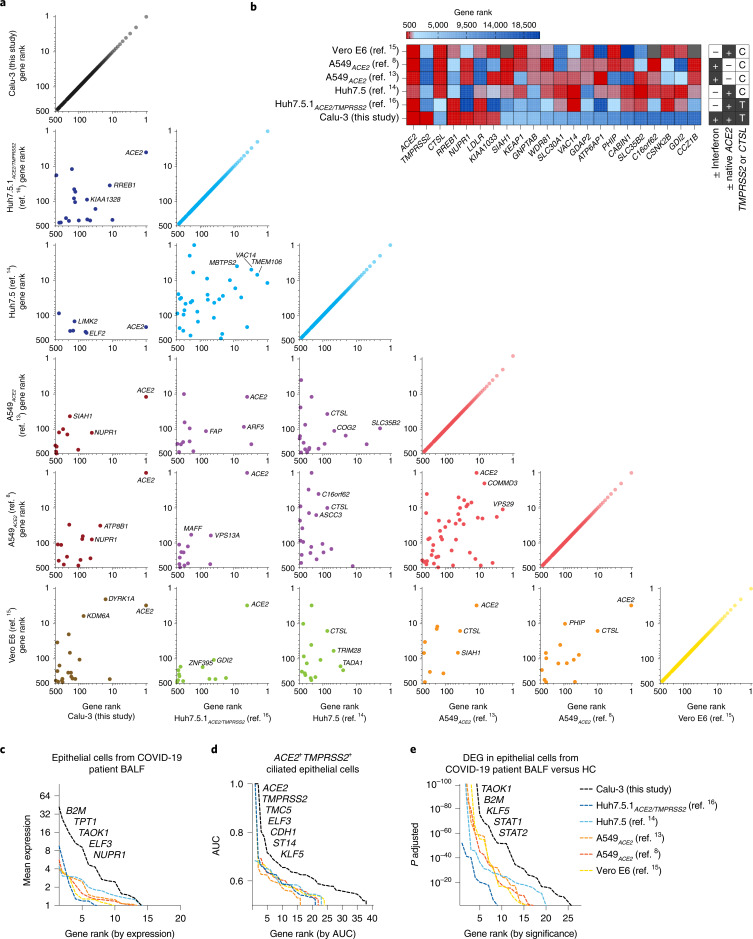
Comparative analysis of LOF screens reveals cell type-specific host factor landscapes of SARS-CoV-2. **a**, Correlation plots of top 500 hits ranked by RRA score from this study and previously reported SARS-CoV-2 KO screens performed in different cell lines. Plots are color-coded by cell line. Black: Calu-3 (human epithelial lung adenocarcinoma cell line), blue: Huh7.5 and Huh7.5.1_ACE2/TMPRSS2_ (human hepatocyte-derived cell lines), red: A549_ACE2_ (ACE2-overexpressing human lung adenocarcinoma cell line), yellow: Vero E6 (African Green Monkey kidney epithelial cell line). The top three overlapping genes with the lowest sum of rank position are displayed. **b**, Heat map indicating screen rank of key SARS-CoV-2 entry factors (left) and all other top 500 ranked hits present in at least three screens (right). Matrix (right) denotes cell line properties. **c**, Top 100 hits across LOF screens ranked by their expression levels in lung epithelial cells from COVID-19 patient BALF scRNA-seq data from Liao et al.^42^. **d**, Top 100 hits across LOF screens ranked by area under the curve (AUC) value for *ACE2*^+^*TMPRSS2*^+^ ciliated human lung epithelial cells based on scRNA-seq meta-analysis, data from Muus et al.^43^. **e**, Top 100 hits across LOF screens ranked based on differential expression in lung epithelial cells from COVID-19 patients compared to healthy individuals, data from Liao et al.^42^. DEG, differentially expressed genes.

We next conducted a comparative analysis of our LOF screen hits with other recent LOF studies of SARS-CoV-2 proviral host factors in different cell lines^8,13–16^. Other than *ACE2*, we noticed surprisingly low overlap in the top 100 hits, with only between zero and four genes overlapping in pairwise comparisons (Fig. 3a). Comparing across all screens, we found that the cell type chosen for infection is the dominant factor determining screen hit overlap, rather than differences in viral strain, MOI or timeline of genomic DNA harvest. A large cluster of gene hits enriched across at least three other screens—but not our Calu-3 screen—include genes encoding vacuolar-associated proteins important for endolysosomal trafficking (*CTSL*, *ATP6AP1*, *GNPTAB*, *VAC14*, *WDR81*, *GDI2* and *CCZ1B*) (Fig. 3b). By contrast, members of the AP1 clathrin adapter complex were uniquely identified in our screen as well as in another recently reported Calu-3 screen (Fig. 2a)^41^. This discrepancy likely highlights differences between the CTSL- and TMPRSS2-dependent entry and trafficking routes of SARS-CoV-2. Taken together, host factors regulating endosomal maturation and CTSL function may be dispensable for SARS-CoV-2 infection when TMPRSS2 is present.

To assess the physiological relevance of our screen hits, we conducted comparative analyses of two distinct scRNA-seq studies: a dataset of human bronchoalveolar lavage fluid (BALF) from COVID-19 patients and controls^42^ and a recent meta-analysis of healthy human lung cell types^43^. First, we identified differentially expressed genes in lung epithelial cells of COVID-19 patients relative to controls and compared these with the top 100 ranked genes from the screens described above. A greater fraction of our Calu-3 screen hits was expressed at higher levels in COVID-19 patients, compared with gene hits from other screens (Fig. 3c). These include genes involved in inflammatory responses (*B2M*, *TAOK1*) and cell proliferation (*ELF3*, *TPT1* and *NUPR1*).

Next, we filtered these RNA-seq datasets to specifically analyze ciliated epithelial cells co-expressing *ACE2* and *TMPRSS2*, presumed to be primary cellular targets of SARS-CoV-2 infection in humans^11,44–46^. We again observed greater enrichment of our Calu-3 gene hits compared with other screens, implying that Calu-3 cells may be more representative of physiologically relevant *ACE2*^+^/*TMPRSS2*^+^ cells in human lungs compared with cell lines employed in other screens (Fig. 3d). Top gene hits found in our Calu-3 screen that were specific to *ACE2*^+^/*TMPRSS2*^+^ ciliated lung cells include regulators of cell proliferation and migration (*TMC5*, *ST14*, *KLF5*) and cell–cell adhesion (*CDH1*) (Fig. 3d). Finally, comparing gene expression profiles from healthy controls and COVID-19 patients’ BALF, we observed that expression of our top Calu-3 gene hits was the most highly modulated upon SARS-CoV-2 infection in the epithelial cell fraction compared with gene hits from other screens (Fig. 3e). Taken together, our analyses uncover strong cell type-specific host factor requirements, with expression of *TMPRSS2* playing a major differentiating role to determine which pathways are important for viral infection. In addition, screen hits identified in Calu-3 cells were more enriched within physiologically relevant human lung epithelial cells.

## Analysis of host restriction factors against SARS-CoV-2

**Fig. 4:**
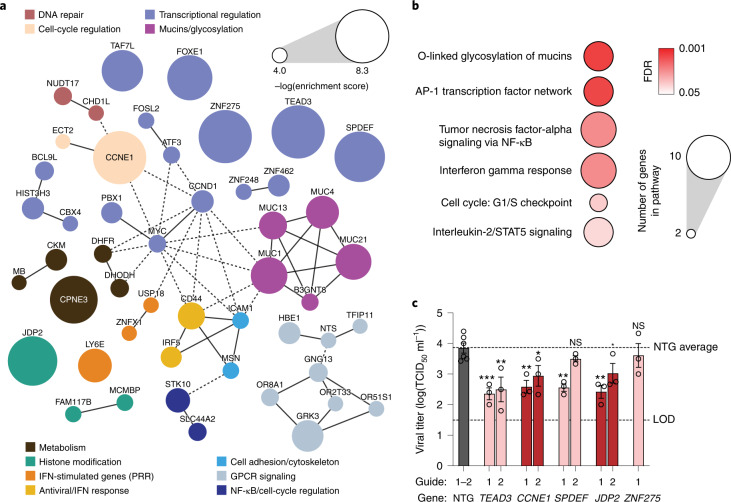
Host restriction factors and pathways of SARS-CoV-2 in lung cells revealed by GOF screening. **a**, Protein–protein interaction network for top 100 enriched hits identified in the CRISPR GOF screen based on STRING analysis. Solid lines between genes indicate direct interaction, dashed lines indicate indirect connections. Nodes are color-coded by functional groups and scaled according to screen enrichment RRA score. GPCR, G-protein-coupled receptor; PRR, pattern recognition receptor. **b**, Pathway analysis of top 100 enriched hits indicates significantly overrepresented pathways with putative antiviral roles. Circle size indicates the number of genes within each pathway, color indicates FDR of pathway enrichment. **c**, Individual validation of the effect of transcriptional upregulation of the top five enriched putative antiviral hits from the GOF screen, measuring SARS-CoV-2 viral titer by TCID_50_. Error bars denote mean ± s.e.m., *n* = 3 biological replicates. The dotted lines indicate the LOD of the assay as well as the NTG average. Significance was determined with individual two-sided *t*-tests between each GOF line and NTG. NS, not significant; *P < 0.05, **P < 0.01, ***P < 0.001, ****P < 0.0001.

**Extended Data Fig. 5:**
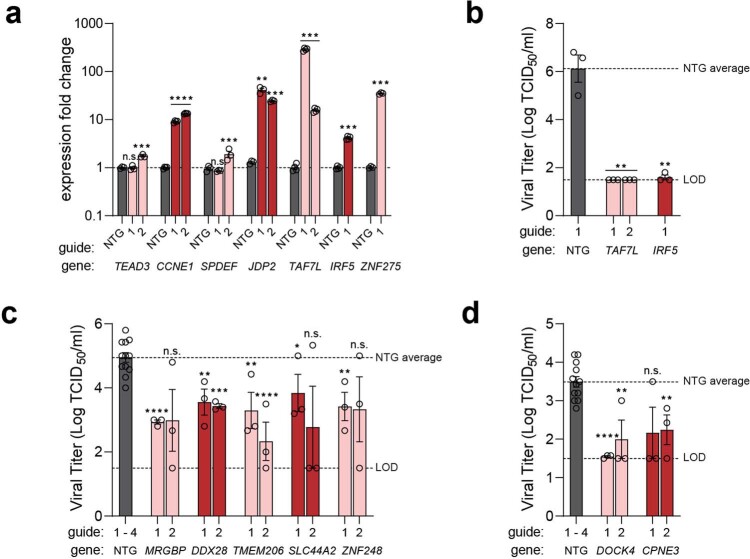
Validation of additional SARS-CoV-2 restriction factors enriched in the GOF screen. **a**. RT-qPCR analysis of target-gene expression for indicated GOF cell lines graphed as expression fold change over NTG for each gene. Statistical significance is measured by individual two-sided t-tests comparing GOF lines to NTG, n = 3 biological replicates. Dotted line indicates NTG expression level (normalized to 1). **b-d**. Viral titer in GOF cell lines infected with SARS-CoV-2 at an MOI of 0.05 for 48 hours and measured by TCID_50_ assay. Dotted line indicates the limit of detection (LOD) of the assay, as well as the NTG average. Statistical significance is measured by individual two-sided t-tests comparing GOF lines to NTG, n = 3 biological replicates. **For all panels**: error bars denote mean ± s.e.m., significance is indicated as: n.s.=not significant, *= P < 0.05, **= P < 0.01, ***=P < 0.001, ****= P < 0.0001.

Our GOF screen gives us the opportunity to systematically investigate host antiviral factors: genes that restrict viral replication when overexpressed. Pathway and protein interaction network enrichment analysis highlighted inflammatory signaling, G-protein-coupled receptor (GPCR) signaling, transcriptional regulation, cell-cycle regulation and mucin glycosylation as key antiviral pathways emerging from our screen results (Fig. 4a,b). We selected our top five enriched GOF hits as well as a representative set of other hits across these pathways, including genes encoding the G1–S checkpoint regulator *CCNE1*, diverse transcriptional regulators (*TEAD3*, *ZNF275*, *SPDEF*, *JDP2*, *TAF7L*, *ZNF248*, *MRGBP*), host helicases (*DDX28*), ion channels (*TMEM206*, *SLC44A2*), modulators of cell–cell junctions (*DOCK4*) and membrane-binding proteins (*CPNE3*) (Extended Data Fig. 5a). TCID_50_ assays of these GOF Calu-3 cell lines infected with SARS-CoV-2 demonstrated reduced viral titers compared with NTG controls to differing extents, confirming an antiviral role for many of these hits. However, upregulation of *ZNF275* had no significant impact on viral load (Fig. 4c and Extended Data Fig. 5b–d). We additionally characterized the extent of target upregulation using quantitative PCR with reverse transcription (RT-qPCR) (Extended Data Fig. 5a), which confirmed significant upregulation in all cases except for guide 1 for *SPDEF* and *TEAD3*. Successful *SPDEF*- and *TEAD3*-mediated viral restriction by TCID_50_ assay for these guides in the absence of a detectable increase in gene expression may be explained by the later RT–qPCR time point relative to the TCID_50_ assay, as the lentivirally integrated GOF components may have been silenced.

The antiviral effect of upregulating *CCNE1*, encoding a protein that drives cells into S phase, is consistent with the observation that upregulation of *CDKN2B*, encoding a cell-cycle inhibitor, increases viral replication (Extended Data Fig. 3f). Coronavirus regulation of the cell cycle by modulating interactions between cyclins and CDKs has been previously shown for SARS-CoV-1, murine hepatitis virus, porcine epidemic diarrhea virus and infectious bronchitis virus, and is a consistent strategy for rerouting host resources toward the coronavirus lifecycle^31^. *TEAD3* and *JDP2* may also regulate cell proliferation in ways that are detrimental to the virus^47,48^. Another GOF-enriched transcription factor, *SPDEF*, is critical for differentiation of goblet cells, a specialized cell type involved in mucus secretion^49^.

Several of our other GOF-enriched hits are known to interact with SARS-CoV-2 viral proteins. The DDX28 helicase interacts with the SARS-CoV-2 N protein and was previously reported to be a protective factor in an RNA interference screen for West Nile virus host factors^50,51^ (Extended Data Fig. 5c). CPNE3, a phospholipid-binding protein, has been implicated as an interaction partner with SARS-CoV-1 nsp1 and viral RNA^52,53^ (Extended Data Fig. 5d). The transcription factor IRF5 has a well-established antiviral role in interferon signaling (Extended Data Fig. 5b). Other interferon-stimulated genes identified in the GOF screen were also predicted to result in an antiviral state, including the lymphocyte antigen 6 family member (*LY6E*), previously reported to antagonize SARS-CoV-2 entry, a ubiquitin-specific peptidase (*USP18*) and the putative mitochondrion-localized dsRNA-sensor *ZNFX1* (Fig. 1d)^54–56^.

Finally, we identified mucin glycosylation as a prominent antiviral pathway impacting SARS-CoV-2 infection (Fig. 4a,b). Mucins comprise a family of high molecular weight, heavily *O*-glycosylated glycoproteins and are the primary constituent of mucus lining the epithelial tract of the lungs and gut^57^. Membrane-anchored mucins (MUC1, MUC4, MUC13, MUC21) form a large, interconnected network in our GOF-enriched protein network analysis, along with the acetylglucosaminyltransferase (B3GNT6) and cell-surface proteins CD44 and ICAM1 also implicated in our screens (Fig. 4a). We decided to investigate membrane-anchored mucins further because of their lung localization, significance of enrichment as an antiviral pathway and lack of previous studies in the context of SARS-CoV-2 infection.

## Membrane-anchored mucins restrict infection of SARS-CoV-2

**Fig. 5:**
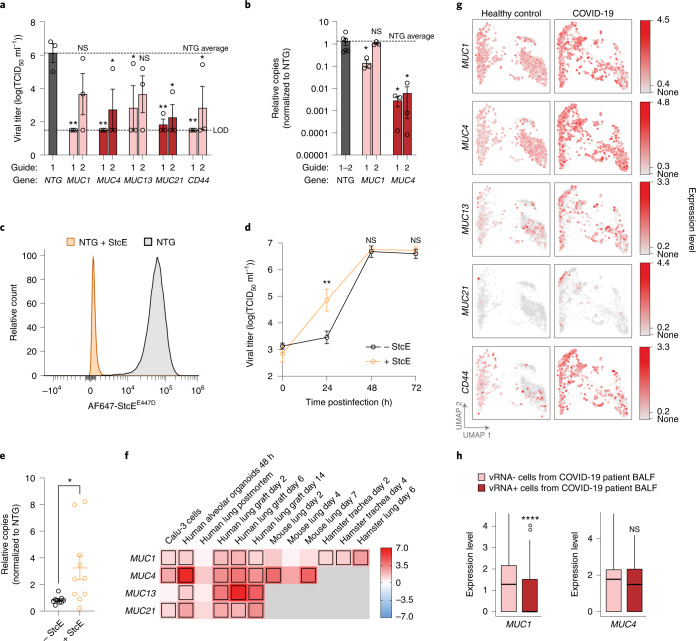
Membrane-tethered mucins are SARS-CoV-2 host restriction factors and upregulated in response to infection. **a**, Individual validation of mucin GOF Calu-3 cells infected with SARS-CoV-2 at an MOI of 0.05 for 48 h, measured by TCID_50_ assay. Two separate sgRNAs were tested per gene. Dotted lines indicate the LOD of the assay (lower) as well as the NTG average (upper). Significance is measured by individual two-sided *t*-tests between each GOF line and NTG, *n* = 3 biological replicates. **b**, Quantification of relative levels of viral N gene copies by RT–qPCR for *MUC1* and *MUC4* GOF lines. Significance was measured by individual two-sided *t*-tests between each GOF line and NTG, *n* = 3 biological replicates. **c**, Flow cytometry of NTG-transduced Calu-3 cells treated with 5 µg ml^−1^ mucin-selective protease (StcE); +StcE *n* = 21,569 cells and −StcE *n* = 27,310 cells. Total mucin levels were detected with an enzyme-inactive form of StcE conjugated to Alexa Fluor 647. **d**, Multistep growth curves of StcE-digested and untreated Calu-3 cells infected with SARS-CoV-2, as measured by TCID_50_ assay. Two-sided *t*-test performed between +StcE and −StcE at each time point, *n* = 3 biological replicates. **e**, RT–qPCR measuring relative levels of viral N gene copies, 24 h postinfection in primary NHBE with and without pretreatment with StcE. Two-sided *t*-test performed between cells ± pretreatment with StcE, *n* = 10 biological replicates. **f**, Heat map representing differential mucin expression levels in cell models, animal models and human lung tissue following infection with SARS-CoV-2. Boxes indicate significant differential expression at FDR < 0.05. Color scale indicates log_2_(fold change) of transcript expression levels after SARS-CoV-2 infection compared with uninfected controls. **g**, UMAP plots of scRNA-seq data for antiviral host factor expression in lung epithelial cells isolated from BALF of COVID-19 patients and healthy individuals from Liao et al.^42^. Cells are colored based on relative expression levels for each gene. All genes shown exhibit differential expression with FDR < 0.001. **h**, Expression of *MUC1* and *MUC4* in human epithelial progenitor cells from BALF of severe COVID-19 patients^42^, comparing gene expression in cells with detected viral RNA (vRNA+, *n* = 282) versus cells without detected viral RNA (vRNA−, *n* = 9,063) in the sample. Cells infected by SARS-CoV-2 were identified using Viral-Track (https://github.com/PierreBSC/Viral-Track) and analyzed by Mann–Whitney *U* test across all panels. Upper and lower hinges correspond to the 75th and 25th percentiles, the center line corresponds to the median and whiskers extend to the most extreme point no further from the closest hinge than 1.5× the interquartile range. Error bars denote mean ± s.e.m. NS, not significant; *P < 0.05, **P < 0.01, ***P < 0.001, ****P < 0.0001.

**Extended Data Fig. 6:**
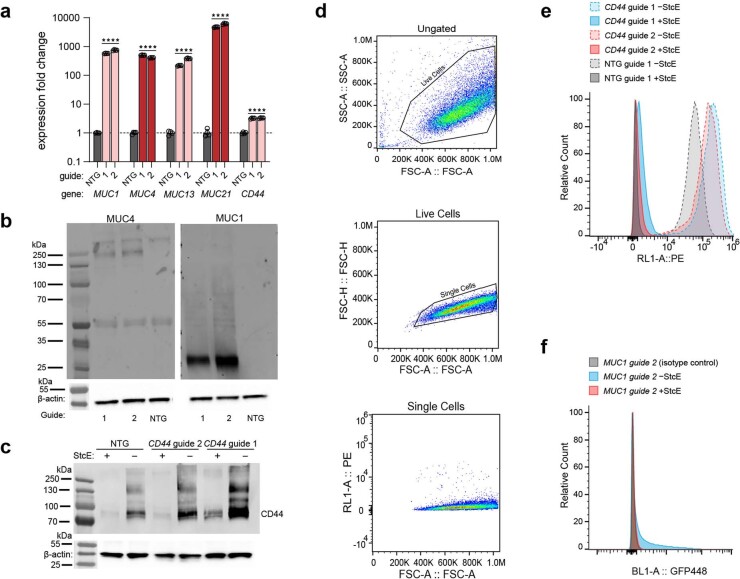
Calu-3 GOF cells express mucins that are sensitive to StcE digestion. **a**. RT-qPCR analysis of target-gene expression for indicated GOF cell lines graphed as expression fold change over NTG for each gene. Statistical significance is measured by individual two-sided t-tests comparing GOF lines to NTG, n = 4 biological replicates. Dotted line indicates NTG expression level (normalized to 1). **b**. Western blot analysis of *MUC1*-, and *MUC4*-overexpressing Calu-3 cells. Bottom panels were imaged in a different channel for β-actin content as a loading control. One western blot was conducted in this experiment. **c**. Western blot analysis of *CD44*-overexpressing Calu-3 cells confirms protein upregulation. Treatment with StcE protease eliminates larger CD44 isoforms; two western blots were conducted with similar results. Bottom panels were imaged in a different channel for β-actin content as a loading control. One western blot was conducted in this experiment. **d**. Gating strategy for flow cytometry analysis, showing NTG Calu-3 cells treated with StcE protease. **e**. Flow cytometry histogram of *CD44*-GOF Calu-3 cells treated with StcE protease, stained with anti-CD44 antibody conjugated to PE. One experiment was conducted, *CD44* guide 1 +StcE n = 12548, -StcE n = 21310, *CD44* guide 2 +StcE n = 11296, -StcE n = 14280. **f**. Flow cytometry histogram of *MUC1* GOF cells treated with StcE protease and stained with anti-MUC1 antibody demonstrates removal of MUC1 on the cell surface by StcE treatment. One experiment was conducted, *MUC1* guide 2 isotype control n = 62687, and *MUC1* guide 2 +StcE n = 33825, and -StcE n = 35500. **For all panels**: error bars denote mean ± s.e.m., significance is indicated as: n.s.=not significant, *= P < 0.05, **= P < 0.01, ***=P < 0.001, ****= P < 0.0001. Source data

To confirm that upregulation of the membrane-tethered mucins is sufficient to reduce SARS-CoV-2 infection, we generated and confirmed individual GOF cell lines for *MUC1*, *MUC4*, *MUC13* and *MUC21* in Calu-3 cells. We also produced a GOF line for the transmembrane glycoprotein CD44, which interacts with MUC1 and can be variably spliced to contain a mucin-like domain (Extended Data Fig. 6a–c)^58,59^. We found that at least one guide per gene in these GOF cell lines exhibited reduced SARS-CoV-2 viral loads compared with NTG controls (Fig. 5a,b).

To determine whether endogenous levels of membrane-anchored mucins could modulate SARS-CoV-2 infection, we treated parental Calu-3 cells with secreted protease of C1 esterase inhibitor (StcE), a bacterial protease that selectively cleaves mucins^60^. We confirmed the specificity of StcE digestion using western blot and flow cytometry for decreased levels of CD44 and MUC1, and showed depletion of StcE binding sites by treatment with a fluorescently labeled enzymatically inactive form of StcE (Fig. 5c and Extended Data Fig. 6c–f)^58,59,61^. Multistep growth curves of SARS-CoV-2 in StcE-treated Calu-3 or primary normal human bronchial epithelial (NHBE) cells revealed a significant increase in viral titer 24 h postinfection relative to untreated controls, indicating that lung cells lacking endogenous mucins are more permissive to viral infection (Fig. 5d,e).

**Extended Data Fig. 7:**
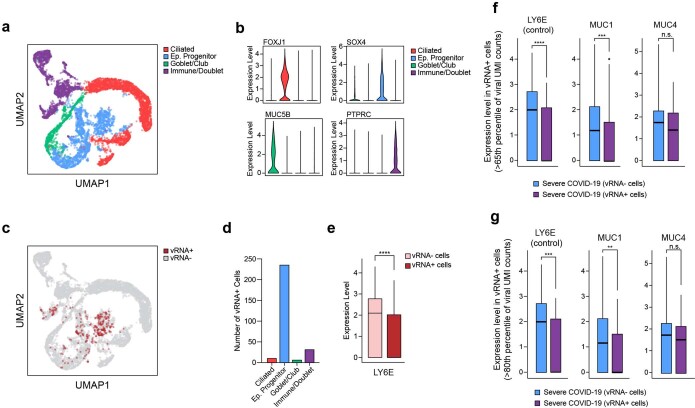
Gene expression analysis of infected versus bystander single cells. **a**. scRNA-seq profiles of epithelial cells from Liao et al.^42^. Cells are colored by assigned epithelial cell type. **b**. Expression of selected marker genes for each cell type. **c**. Identification of infected cells using Viral-Track^67^. Any cell with at least one detected high-confidence viral transcript is classified as positive. **d**. Number of viral RNA positive cells (vRNA+) by cell type. **e**. As a positive control for our methodology, expression of LY6E, a previously identified antiviral factor and GOF-enriched hit in our screen, is shown in epithelial progenitor cells comparing levels in vRNA+ cells (n = 282) and vRNA- cells (n = 9063). Indicated comparison was made by Mann-Whitney U test. **f-g**. Comparison of gene expression in viral RNA positive (vRNA+) and viral RNA negative (vRNA-) cells. (**f**) Viral UMI cutoffs of >65% of all cells were used to identify vRNA+ cells (vRNA + , n = 94 cells and vRNA-, n = 9251 cells). (**g**) Same as (f) except a viral UMI cutoff of >80% was used. (vRNA + , n = 48 cells, and vRNA- n = 9297 cells). Indicated comparisons were made by Mann-Whitney U test. **For all panel**s: *= p < 0.05, **= p < 0.01, ***= p < 0.001, ****= p < 0.0001. Box plots: Upper and lower hinges correspond to the 75th and 25th percentiles, center line corresponds to the median, and whiskers extend to the most extreme point no further from the closest hinge than 1.5 * the interquartile range.

We next assessed mucin gene expression in RNA-seq datasets of lung tissues to determine whether membrane-anchored mucins are regulated in response to SARS-CoV-2 infection. Across six different model systems in addition to postmortem human lung tissue, we observe consistent upregulation of mucins from 2 days up to 2 weeks postinfection (Fig. 5f)^4,43,62–64^. Whereas hamster and mouse models only exhibited significant upregulation of an individual mucin (*MUC1* and *MUC4*, respectively), human alveolar organoids in vitro and human lung grafts in vivo upregulated all four membrane-tethered mucins. We further observed that the epithelial cell fraction of COVID-19 patients’ BALF revealed significant upregulation of all four transmembrane mucins in addition to *CD44* (Fig. 5g). Binning individual cells based on the presence (vRNA+) or absence (vRNA–) of SARS-CoV-2 RNA, we show that *MUC1* was expressed at lower levels in SARS-CoV-2-positive versus SARS-CoV-2-negative epithelial progenitor cells in COVID-19 patients, consistent with a protective role (Fig. 5h and Extended Data Fig. 7). Overall, these gene expression profiles suggest mucin upregulation is a broad antiviral host response across multiple species.

**Fig. 6:**
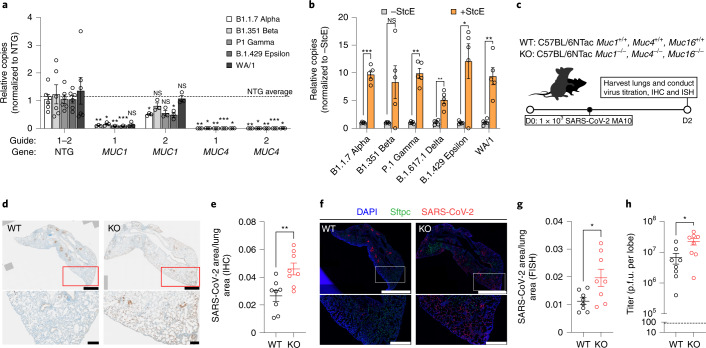
Membrane-tethered mucins restrict infection of SARS-CoV-2 variants in vitro and mouse-adapted SARS-CoV-2 in vivo. **a**, RT–qPCR measuring relative levels of viral N gene copies in mucin-overexpressing Calu-3 cells infected with the indicated SARS-CoV-2 variants at an MOI of 0.05 for 24 h. The dotted line indicates the NTG average for two separate guides each with *n* = 3 biological replicates. A two-tailed *t*-test was performed for each viral variant relative to its own NTG control. **b**, SARS-CoV-2 infection of WT Calu-3 cells with or without pretreatment with StcE across variants. Two-sided *t*-test performed for +StcE versus −StcE for each viral variant, *n* = 5 biological replicates. **c**–**h**, *Muc1*^−*/*−^/*Muc4*^−*/*−^/*Muc16*^−*/*−^ triple KO and control mice were infected with 10^3^ p.f.u. of mouse-adapted SARS-CoV-2 intranasally and viral loads were analyzed in the lungs 2 d postinfection. **c**, Schematic depicting experimental conditions for in vivo infection, and subsequent analysis of SARS-CoV-2 infection in either WT or mucin KO mice. **d**, IHC staining for SARS-CoV-2 nucleocapsid followed by DAB development and hematoxylin II staining on paraffin-embedded lung sections. Shown are representative lungs from *n* = 8 mice. Scale bars, 1 mm (upper) and 100 µm (lower). **e**, Quantification of **d**. A two-tailed *t*-test was performed between mucin WT and KO groups, *n* = 8 mice. **f**. RNA-ISH probing for SARS-CoV-2 RNA on paraffin-embedded lung sections. Shown are representative lungs from *n* = 8 mice. Scale bars, 2 mm (upper) and 500 µm (lower). DAPI, 4,6-diamidino-2-phenylindole. **g**, Quantitation of **f**. A two-tailed *t*-test was performed between mucin WT and KO groups, *n* = 8 mice. **h**, Quantitation of infectious viral particles per lung lobe by plaque assay from *n* = 8 mice. A two-tailed *t*-test was performed between mucin WT and KO groups. Error bars denote mean ± s.e.m. NS, not significant; *P < 0.05, **P < 0.01, ***P < 0.001, ****P < 0.0001.

To determine the impact of membrane-anchored mucins on infection of diverse SARS-CoV-2 clinical isolates, we infected our *MUC1* and *MUC4* GOF Calu-3 lines with alpha (B.1.1.7), beta (B.1.351), gamma (P.1), epsilon (B.1.429) and WA/1 variants. We found that overexpression of either *MUC1* or *MUC4* restricted viral replication of diverse SARS-CoV-2 variants relative to NTG controls (Fig. 6a). Next, StcE treatment followed by infection with these same variants, in addition to the delta (B.1.617.1) variant, rendered Calu-3 cells significantly more permissive to viral infection, with the exception of B1.351 (Fig. 6b). Taken together, these data suggest that membrane-anchored mucins restrict infection of multiple SARS-CoV-2 variants.

Next, we asked whether membrane-anchored mucins were protective against SARS-CoV-2 infection in vivo. Given that *MUC1* and *MUC4* emerged from our GOF screens, but not our LOF screens, and that these two mucins are both highly expressed in mouse and human lung epithelial cells (Fig. 5g), we reasoned that their roles in restricting SARS-CoV-2 infection may exhibit redundancy. To investigate multiplexed membrane-anchored mucin KOs, we infected a triple membrane-anchored mucin KO mouse (*Muc1*^−*/*−^/*Muc4*^−*/*−^/*Muc16*^−*/*−^) with 10^3^ plaque-forming units (p.f.u.) of a mouse-adapted strain of SARS-CoV-2 (MA10). We compared viral loads in the lungs of these mice with wild-type (WT) control animals, harvesting lungs at 2 d postinfection (Fig. 6c). Immunohistochemistry (IHC) staining for SARS-CoV-2 N protein and RNA in situ hybridization (RNA-ISH) targeting SARS-CoV-2 RNA revealed greater levels of viral antigen (Fig. 6d,e) and viral RNA (Fig. 6f,g) in the lungs of triple mucin KO mice, which also displayed higher viral titers compared with WT (Fig. 6h). Consistent with our in vitro data, membrane-tethered mucins are protective against SARS-CoV-2 infection in vivo.

## Mucins modulate cell entry of SARS-CoV-2

**Fig. 7:**
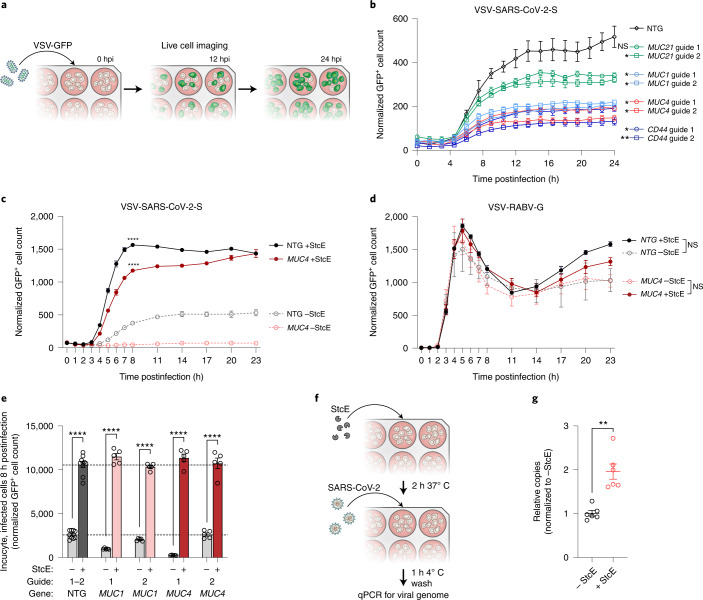
Membrane-tethered mucins restrict SARS-CoV-2 Spike-mediated entry. **a**, Schematic of the pseudotype entry assay using a VSV-CoV-2-S encoding GFP. Following infection, cells are imaged regularly over 24 h to track viral infection by quantifying GFP^+^ cell counts. hpi, hours postinfection. **b**, Time course of VSV-CoV-2-S infection of NTG, *MUC1, MUC4, MUC21* and *CD44-*overexpressing GOF cell lines. The significance of the difference relative to NTG at 24 h postinfection was determined by two-sided *t*-test, *n* = 3 biological replicates. **c**., Time course of VSV-CoV-2-S infection of NTG and *MUC4* GOF cells pretreated with StcE mucinase before VSV-CoV-2-S infection. Significance of the difference between StcE-treated cells versus untreated cells for each cell line at the point of saturation at 8 h postinfection was determined by two-sided *t*-test, *n* = 3 biological replicates. **d**, Time course of VSV-RABV-G infection in NTG and *MUC4* GOF cells pretreated with StcE before pseudotype virus infection. VSV-RABV-G, VSV particles pseudotyped with rabies virus glycoprotein. Significance comparing StcE-treated versus untreated cells at 5 h postinfection was determined by two-sided *t*-test, *n* = 3 biological replicates. **e**, Pseudotype entry assay measuring GFP^+^ cells at 8 h postinfection comparing infection rates between NTG or mucin-overexpressing cells with and without StcE pretreatment. The full dataset is displayed in Extended Data Fig. 9. *n* = 5 biological replicates; two-tailed *t*-tests were performed comparing indicated pairs. The dotted line indicates the NTG average for two separate guides, with and without StcE treatment. **f**, Schematic depicting a SARS-CoV-2 binding assay. **g**, RT–qPCR of relative levels of viral N gene copies following a virus binding assay conducted on Calu-3 cells at an MOI of 2.5 with or without StcE treatment. Significance comparing StcE-treated cells versus untreated cells was determined by two-sided *t*-test, *n* = 6 biological replicates. Error bars denote mean ± s.e.m. NS, not significant; *P < 0.05, **P < 0.01, ***P < 0.001, ****P < 0.0001.

We next sought to identify the stage of the viral life cycle that is affected by mucin upregulation. Because all four mucins enriched in our GOF screen are expressed at the host cell surface, we hypothesized that they reduce SARS-CoV-2 entry. Indeed, live imaging of cells infected with our VSV-CoV-2-S pseudotype virus demonstrated that CRISPR-mediated overexpression of all tested membrane-tethered mucins, including *CD44*, inhibited spike-mediated viral entry relative to NTG control, in agreement with our viral titer validation data (Fig. 7a,b). The StcE-mediated digestion of extracellular mucins in *MUC4* and *MUC1* GOF as well as NTG cell lines led to a dramatic increase in VSV-CoV-2-S infection compared with untreated cells, indicating that mucin removal renders cells more permissive to VSV-CoV-2-S infection, consistent with our in vivo data (Fig. 7c,e and Extended Data Fig. 9a). By contrast, endogenous expression or CRISPR-mediated overexpression of *MUC4* in Calu-3 cells did not have an effect on VSV-RABV-G infection, suggesting that virus-specific interactions may govern the antiviral effect of mucins during viral entry (Fig. 7d).

**Extended Data Fig. 8:**
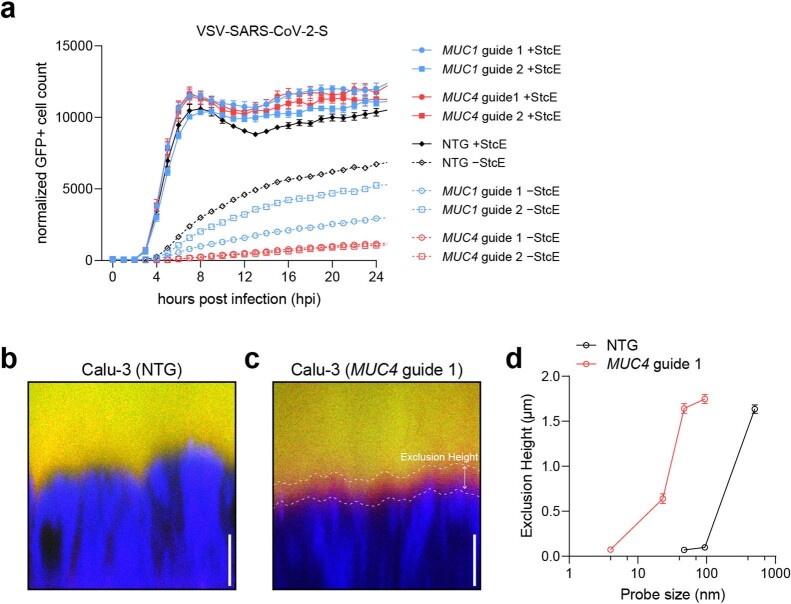
Membrane-tethered mucins restrict SARS-CoV-2 entry and *MUC4* GOF Calu-3 cells possess a denser glycocalyx. **a**. Time course tracking infection levels of VSV-CoV-2-S encoding GFP. Infection of NTG, and *MUC1* or *MUC4* overexpression cell lines with and without StcE treatment, n = 5 biological replicates. The above Incucyte trace is the full dataset for the summary displayed in Fig. 7E. **b-c**. A glycocalyx density assay measuring the mesh size of the Calu-3 glycocalyx on (b) NTG or (c) *MUC4* GOF lines. The assay uses a live-cell two-color fluorescent imaging approach using two types of probes of known sizes: green fluorescent test probes of various sizes as well as Texas-Red dextran with a known size of 2 nm^68^. Scale bars indicate 5 µm. **d**. Quantification of probe exclusion by the Calu-3 glycocalyx. For each sized probe n = 5 biological replicates (with the exception of the NTG 500 nm probe, with n = 3). **For all panels**: error bars denote mean ± s.e.m., significance is indicated as: n.s.=not significant, *= P < 0.05, **= P < 0.01, ***=P < 0.001, ****= P < 0.0001.

Given previous literature reporting a steric hindrance role of membrane-anchored mucins in inhibiting Influenza A virus infection^65^, we hypothesized that membrane-anchored mucins block binding of SARS-CoV-2 virions to cells. To test this, we conducted a SARS-CoV-2 binding assay on StcE-treated or control Calu-3 cells, which revealed a significantly higher level of bound virions to StcE-digested compared with undigested cells (Fig. 7f,g). Consistent with this steric hindrance hypothesis, we found that *MUC4* GOF cells had a denser glycocalyx layer compared with control cells, as measured by cell-surface exclusion of different sized, fluorescently labeled dextran probes (Extended Data Fig. 8b–d). Taken together, our data indicate that endogenous and upregulated membrane-tethered mucins restrict SARS-CoV-2 entry, specifically at the step of initial cell binding.

**Extended Data Fig. 9:**
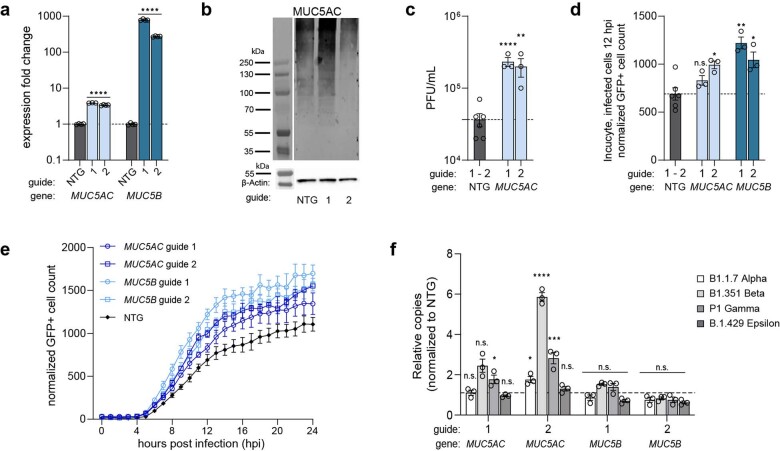
Gel-forming mucins promote SARS-CoV-2 entry. **a**. RT-qPCR analysis of target-gene expression for indicated GOF cell lines graphed as expression fold change over NTG for each gene. Statistical significance is measured by individual two-sided t-tests comparing GOF lines to NTG, n = 4 biological replicates. Dotted line indicates NTG expression level (normalized to 1). **b**. Western blot analysis of *MUC5AC* GOF Calu-3 cells. Bottom panel was imaged in a different channel or lanes were cropped out between the ladder and target lanes (upper left). One western blot was conducted in this experiment. **c**. Plaque assay on the indicated Calu-3 GOF cells infected with SARS-CoV-2 for 24 hours at an MOI of 0.1. Significance was determined by two-sided t-tests comparing individual GOF cell lines to NTG, n = 3 biological replicates. **d**. Pseudotype-entry assay measuring GFP + cells at 12 hpi with VSV-CoV-2-S (SARS-CoV-2 Spike-pseudotyped Vesicular stomatitis virus encoding GFP). Significance was determined by two-sided t-tests comparing individual GOF cell lines to NTG, n = 3 biological replicates. Dotted line indicates the NTG average. **e**. Incucyte trace tracking GFP^+^ (VSV-CoV-2-S infected) cells from the same experiment shown in (**d**). **f**. RT-qPCR analysis of SARS-CoV-2 N gene copies 24 hpi of four different SARS-CoV-2 variants in the indicated mucin GOF Calu-3 cells, graphed as normalized to NTG average (indicated by a dotted line). Significance was determined by individual two-sided t-tests comparing the GOF mucin line to NTG, n = 3 biological replicates. **For all panels**: error bars denote mean ± s.e.m., significance is indicated as: n.s.=not significant, *= P < 0.05, **= P < 0.01, ***=P < 0.001, ****= P < 0.0001. Source data

Mucins are divided into two distinct classes: membrane-anchored mucins such as *MUC1* or *MUC4*; and secreted, gel-forming mucins such as *MUC5AC* and *MUC5B*. Although diverse membrane-anchored mucins emerged from our GOF-enriched screen and were validated to restrict SARS-CoV-2 infection in vitro and in vivo, *MUC5AC* emerged as a hit from our GOF-depleted screen, suggesting a proviral role. To test the effect of *MUC5AC* in modulating SARS-CoV-2 infection, we generated *MUC5AC* and *MUC5B* GOF Calu-3 cells and validated their expression at messenger RNA and protein levels (Extended Data Fig. 9a,b). By contrast to membrane-anchored mucins, these lines did not show any protective effect against SARS-CoV-2 infection. Consistent with its depletion in our GOF screen, *MUC5AC* but not *MUC5B* upregulation increased susceptibility to the SARS-CoV-2 WA/1 clinical isolate and all tested variants except for B.1.429 (Extended Data Fig. 9c–f). The contrasting phenotypes of gel-forming and membrane-anchored mucins highlight the complex roles of mucins during SARS-CoV-2 infection.

## Mucins modulate infection of diverse respiratory viruses

**Fig. 8:**
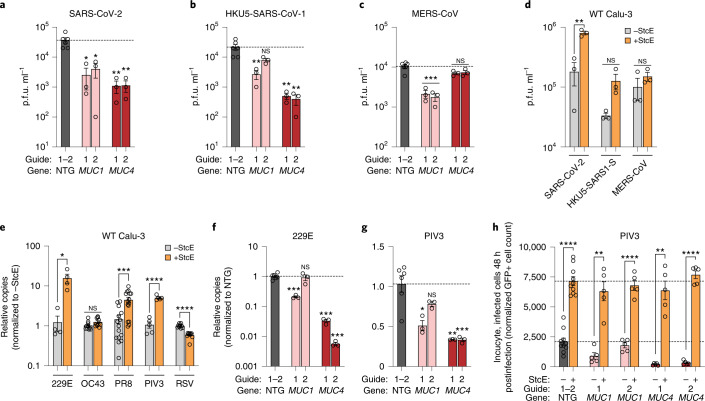
Membrane-tethered mucins restrict infection of multiple respiratory viruses. **a**–**c**, Calu-3 mucin GOF cells were infected with (**a**) SARS-CoV-2, (**b**) HKU5-SARS-CoV-S or (**c**) MERS-CoV at an MOI of 0.1. At 24 h postinfection, viral titers were quantified by plaque assay. The dotted line is the NTG average, *n* = 3 biological replicates. Significance was calculated by a two-sided *t*-test between each GOF cell line versus NTG. **d**, As **a**–**c**, except on WT Calu-3 cells pretreated with StcE. *n* = 3 biological replicates, two-sided *t*-tests were performed for the indicated pairs. **e**, Calu-3 cells were treated with StcE or left untreated and then infected with the indicated virus for 24 h. Relative levels of viral gene copies were quantified by RT–qPCR. Displayed are *n* = 4 (HCoV-229E), *n* = 10 (HCoV-OC43), *n* = 15 (Influenza A virus, PR8), *n* = 5 (PIV3) and *n* = 10 (RSV) biological replicates. Two-sided *t*-tests were performed for the indicated pairs. **f**,**g**. Mucin-overexpressing Calu-3 cells were infected with HCoV-229E or PIV3, and relative levels of viral gene copies were quantified 24 h postinfection by RT–qPCR. *n* = 3 biological replicates, the dotted line is the NTG average. Significance was calculated by a two-sided *t*-test between each GOF cell line versus NTG. **h**, PIV3 infection of the indicated GOF Calu-3 cells, with and without StcE treatment, at 48 h postinfection quantifying GFP^+^ cells. Displayed is a single time point from the experiment shown in Extended Data Fig. 10a. Displayed are *n* = 5 biological replicates, two-sided *t*-tests were performed for the indicated pairs. Dotted lines indicate the NTG average for two separate guides, with (upper) and without (lower) StcE treatment. Error bars denote mean ± s.e.m. NS, not significant; *P < 0.05, **P < 0.01, ***P < 0.001, ****P < 0.0001.

**Extended Data Fig. 10:**
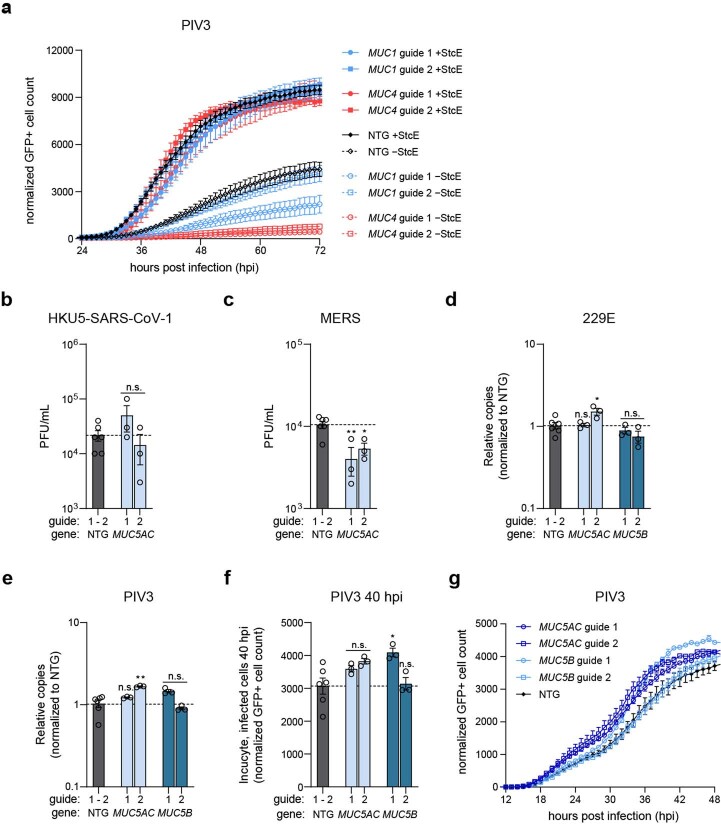
Membrane-tethered and gel-forming mucins modulate infection of multiple respiratory viruses. **a**. Incucyte trace tracking GFP^+^ (PIV3 infected) cells over 72 hours in Calu-3 mucin GOF cell lines, either pretreated with StcE or not, n = 5 biological replicates. Full dataset for the 48-hour time point from Fig. 8h. **b-c**. Plaque assay measuring viral loads of the indicated Calu-3 GOF cells infected with HKU5-SARS-Cov-1, or MERS-CoV, respectively, at 24 hpi at an MOI of 0.1. Dotted line indicates the NTG average for guides tested. Significance was calculated by individual two-sided t-tests comparing each cell line to NTG, n = 3 biological replicates. **d-e**. qRT-PCR measuring viral gene copies 24 hpi in mucin-overexpressing cell lines for 229E (MOI 0.05), or PIV3 (MOI 0.0002), respectively. Dotted line indicates the NTG average. Significance was calculated by individual two-sided t-tests comparing each cell line to NTG, n = 3 biological replicates. **f**. GFP^+^ (infected) cells from indicated GOF Calu-3 cell lines infected with PIV3 at MOI 0.0002 at 40 hpi. Significance was calculated by individual two-sided t-tests comparing each cell line to NTG, n = 3 biological replicates. Dotted line indicates the NTG average.**g**. Incucyte trace tracking GFP + infected cells from (f). **For all panels**: error bars denote mean ± s.e.m., significance is indicated as: n.s.=not significant, *= P < 0.05, **= P < 0.01, ***=P < 0.001, ****= P < 0.0001.

Finally, we investigated whether the antiviral effect we observed for membrane-anchored mucins against SARS-CoV-2 would extend to other respiratory viruses. First, we tested the effect of *MUC1* and *MUC4* GOF on the close relatives MERS-CoV and the bat coronavirus HKU5 pseudotyped with SARS-CoV-1 Spike (HKU5-SARS-COV-1-S). Although SARS-CoV-2 infection was restricted by overexpression of both *MUC1* and *MUC4*, we found that *MUC4* GOF had a more robust impact on HKU5-SARS-COV-1-S infection compared with *MUC1*, whereas the converse was true for MERS-CoV infection (Fig. 8a–c). Further, removal of endogenous mucins by StcE digestion had little impact on HKU5-SARS-CoV-1-S and MERS-CoV infection relative to undigested control cells (Fig. 8d). Extending this analysis to more diverse respiratory viruses, we show that mucin removal led to an increase in infection for influenza virus PR8, consistent with previous reports, as well as human coronavirus 229E (HCoV-229E) and human parainfluenza virus PIV3 (refs. ^65,66^). By contrast, no effect was observed on infection with HCoV-OC43, and a surprising reduction in infection was seen for respiratory syncytial virus (RSV) (Fig. 8e)^65,66^. The protective function of mucins against 229E and PIV3 infection was further confirmed for *MUC1*- and *MUC4*-overexpressing cells (Fig. 8f–h and Extended Data Fig. 10a). Consistent with our observations for SARS-CoV-2, secreted mucins *MUC5AC* and *MUC5B* did not protect against infection for most viruses, with the exception of MERS-CoV (Extended Data Fig. 10b–g). Taken together, these data indicate an important role for mucins across respiratory viruses, with unique functions in promoting or restricting viral infection dependent on the mucin and virus, highlighting the complexity of host–pathogen interactions between respiratory viruses and lung mucins.

## Conclusions

Here, we report the first genome-scale GOF screen exploring host factors influencing SARS-CoV-2 infectivity, in addition to the first LOF screen that exploits canonical ACE2- and TMPRSS2-mediated viral entry into lung epithelial cells (Supplementary Tables 1–3). The combination of bidirectional genome-scale screens enables the assignment of proviral or antiviral roles for individual genes into a systematic functional catalog of unique host factor dependencies. We further observed their robust enrichment in scRNA-seq datasets of healthy and infected human lung epithelium. Finally, our mechanistic investigation into the role of mucins during respiratory virus infection reveals a restrictive role of viral entry of multiple viruses by membrane-tethered mucins. By dissecting the interactions between SARS-CoV-2 and lung epithelial cells, these host factor screens provide a starting point for host-directed intervention and new antiviral therapeutic strategies.

## Methods

### Cell culture

The human lung epithelial cell line Calu-3 (UC Berkeley Cell Culture Facility) was maintained in RPMI media supplemented with GlutaMAX (Thermo Fisher Scientific), 10% fetal bovine serum (FBS, HyClone) and 100 μg ml^−1^ penicillin and streptomycin (Gibco; R10 medium). Cells were grown in T225 flasks (Thermo Fisher Scientific) and regularly passaged at 80–90% confluence with 10× concentrated TrypLE Express (Thermo Fisher Scientific). After lentiviral transductions with Cas9 and dCas9–VP64 constructs, Calu-3 cells were cultured in R10 media additionally supplemented with 16 ng ml^−1^ of hepatocyte growth factor (HGF, Stem Cell Technologies) to preserve viability and support robust growth^67^. NHBE were purchased from Lonza (CC-2541) and cultivated in bronchial epithelial cell growth medium (CC-3170) as per the manufacturer’s instructions.

### Genome-wide, bidirectional CRISPR screens

Calu-3 cells were transduced with lenti Cas9-Blast (Addgene #52962) or with lenti dCAS–VP64_Blast (Addgene #61425), gifts from F. Zhang. Approximately 24 h post-transduction, cells were selected with 10 μg ml^−1^ blasticidin for 10 d. Cas9 and dCas–VP64 Calu-3 knock-in lines were then transduced with Brunello and Calabrese Set A libraries (Addgene #73179 and #92379) as appropriate, which were gifts from J. Doench. Library transductions were conducted to maintain >1,000× representation of sgRNAs. The Brunello library contains 76,441 sgRNAs, so >2.7 × 10^8^ cells were transduced at an MOI of 0.3. The Calabrese library contains 56,762 sgRNAs, so >2 × 10^8^ cells were transduced at an MOI of 0.3. At 24 h postinfection, cells were selected with 1.5 μg ml^−1^ of Puromycin antibiotic for 5 d.

For the LOF screen, the Cas9 knock-in cell line transduced with Brunello sgRNAs were seeded at 3.5 × 10^7^ cells per T225 flask and allowed to grow to 70% confluency. At this point, half of the cells were harvested for a day 0 (D0) time point to serve as a reference for sgRNA enrichment analysis. The remaining cells were infected with the SARS-CoV-2 USA/WA-1 isolate at an MOI of 0.05. Cells were incubated for 4 d until >70% CPE was apparent. Throughout the infection, the medium was replaced every other day with R10 + HGF. For the GOF screen, dCas–VP64 knock-in cells transduced with Calabrese sgRNAs were treated similarly except they were infected at an MOI of 0.05 for 5 d until >70% CPE was apparent. At 5 d postinfection, surviving cells were uplifted and lysed according to the manufacturer’s instructions using a genomic DNA extraction kit, followed by heat inactivation (Zymo Research).

### Next-generation sequencing

Guide RNA cassettes were amplified from extracted genomic DNA to generate Illumina sequencing libraries. Namely, 3 µg of genomic DNA was added per 50 µl PCR reaction using staggered primers to increase base diversity. PCR products were then pooled and purified using QIAquick PCR purification kits (Qiagen). Different libraries were then quantified with Kappaquant to determine relative concentrations of amplified product, then pooled to match concentrations. Pooled samples were sequenced by Illumina Novaseq. In addition to the uninfected D0 and infected time points, the lentiviral plasmid prep was also sequenced to assess any guide distribution skew resulting from transduction.

### Computational analysis of CRISPR screens

A previously published method for CRISPR screen analysis, MAGeCK^68^ was used to rank genes based on redundant targeting guides via robust rank aggregation (RRA). Our LOF screen was performed with three biological replicates and our GOF screen with four biological replicates maintaining >1,000× sgRNA coverage. sgRNA enrichment and depletion were assessed in infected versus D0 uninfected samples for each paired sample using the paired flag. Each set of top 100 screen hits was determined based on MAGeCK RRA rank and the false discovery rate (FDR) in a negative control analysis. To investigate the relationships within each of these sets, we performed protein–protein interaction networks functional enrichment analysis with STRING^69^. Any genes that exhibited depletion or enrichment in the top 500 hits between the plasmid library and the uninfected control were removed from STRING analysis because they are likely confounded by affecting cellular growth or survival unrelated to viral infection. Nodes were resized based on MAGeCK scores. Specifically, the radius (*r*) of each node was calculated as a constant factor (*c*) scaled by 1.5 raised to the z-score (*z*) of the corresponding gene’s negative log MAGeCK score: *r* = *c* × 1.5^*z*^. *z* was calculated from the distribution of the negative log MaGeCK scores in the relevant top 100 gene set.

### Secondary validation with individual guide RNAs

Lentivirus for Cas9 KO was produced in six-well plates. In brief, low-passage HEK293FT cells were grown in DMEM supplemented with 10% FBS (D10 medium) and passaged using TrypLE (Gibco). For viral plasmid transfection, polyethylenimine ‘Max’ (PEI, linear, *M*_r_ 40,000, VWR) at a concentration of 1 mg ml^−1^ and pH 7.1 was used. PEI (0.71 μg PEI per cm^2^ cell culture area) was mixed with 1 ml of DMEM. pMD2.G, PAX and target-guide plasmid (0.178 μg cm^−^^2^, at a pMD2.G/psPAX/guide plasmid ratio of 0.25:0.5:1 were mixed with 1 ml of DMEM. Following 10 min of incubation at room temperature, the DNA and PEI mixtures were mixed and incubated for 30 min at room temperature. Approximately 150,000 cells per cm^2^ of 293FT cells in suspension were mixed with the PEI–DNA DMEM mixture, incubated for 5 min at room temperature, then plated in D10. After 48 h, the viral supernatant was harvested, centrifuged at 800x*g* for 5 min to remove cell debris, and used to transduce 800,000 Cas9-expressing Calu-3 cells. 24 h later, cells were selected with blasticidin. Lentivirus for CRISPR activators was produced as described above, scaled to T75 flasks. Clarified lentiviral supernatant was concentrated using LentiX Concentrator (Takara Bio). The concentrated aptamer guide RNA virus was used to transduce five million Calu-3 cells expressing dCas9–VP64 before blasticidin selection the following day.

### Calu-3 validation infections and TCID_50_ assay

A total of 2 × 10^5^ Calu-3 cells were seeded into 24-well plates and infected with SARS-CoV-2 48 h post-seeding at an MOI of 0.05. Viral inoculums were incubated with cells for 30 min at 37 °C, at which time they were removed, and cells were washed once with 1× PBS. Regular media R10 + HGF was then replaced, and cells were incubated for the times indicated in the figures (24–48 h postinfection). Plates were then frozen to lyse cells and thawed when ready to quantify virus by TCID_50_. To titer infectious viral particles, plates were thawed and viral lysates were serially diluted; each dilution was applied to eight wells in 96-well plates containing Vero E6 cells. Three days later, CPE was determined visually, and TCID_50_ per ml was calculated using the dilution factor required to produce CPE in half of the wells (4/8) for a given dilution.

### SARS-CoV-2 stock preparation and infections

The USA-WA-1/2020 strain of SARS-CoV-2 used was obtained from BEI Resources. The original stock from BEI was passaged through a 0.45 µM syringe filter and then 5 µl was inoculated onto 80% confluent T175 flasks (Nunc) of Vero E6 cells to produce our p1 stock. The CPE was monitored daily, and flasks were frozen when cells exhibited ~70% CPE, ~48–72 h postinfection. Lysates were then thawed, collected and cell debris was spun down at 3,000 r.p.m. for 20 min. The clarified virus-containing supernatants were then aliquoted and infectious viral particles were quantified with a TCID_50_ assay. To produce p2 working SARS-CoV-2 stocks, 5 µl of the p1 stock was inoculated onto 80% confluent T175 flasks of Vero E6 cells as described above. SARS-CoV-2 variant viral stocks were produced as described previously^70^. In brief, the B.1.351 isolate was derived from a nasal swab at Stanford Hospital. Nasal swab medium was inoculated onto Vero E6 cells in the BSL-3 facility at Stanford University. Vero E6 cells were monitored for CPE and harvested as above for p0 stocks. p1 and p2 stocks were generated as above. Evaluation of antiviral activity of chemical compounds was conducted as described previously^71^. In brief, cells were infected in 384-well plates at a MOI of 0.05 in a total volume of 6 μl per well. Cells were harvested and analyzed using CellTiter-Glo 2.0 reagent (Promega) once complete CPE was observed in dimethylsulfoxide-treated infected wells (96 h postinfection for Calu-3). Luminescence was read on a Spectramax L (Molecular Devices). Compound activity was measured in infected cells by comparing ATP levels in dimethylsulfoxide- and compound-treated cells. Cell viability was determined by comparing ATP levels in uninfected cells treated with dimethylsulfoxide and compounds.

### Production and infection of Vero/furin cells

To determine whether our SARS-CoV-2 viral stocks remained responsive to furin after passage in Vero E6 cells, we produced Vero E6 cells overexpressing the human furin gene. To this end, Vero E6 cells were transduced with lentivirus encoding the human furin gene. Cells were then selected with puromycin for three passages in 10 μg ml^−1^ puromycin. Furin-overexpressing cells (Vero/furin) along with control transduced cells (Vero), were then infected with SARS-CoV-2 at an MOI of 0.05 and TCID_50_ was determined on whole-cell lysates 24 h postinfection. To further confirm that our viral stocks were responsive to furin cleavage, we also infected HPMEC/ACE2 (described in Biering et al.^71^), with SARS-CoV-2 at an MOI of 0.05. At 24 h postinfection syncytia (cell–cell fusion) was apparent and evaluated by phase contrast microscopy.

### SARS-CoV-2 stock sequencing

Viral RNA was extracted from working stocks with the Qiagen RNA Easy Minikit (catalog no. 74104). Library preparation was performed by the Functional Genomics Laboratory (FGL), a QB3-Berkeley Core Research Facility at UC Berkeley. Total RNA samples were checked on Bioanalyzer (Agilent) for quality, and only high-quality RNA samples (RNA Integrity Number > 8) were used. At the FGL, SARS-CoV-2 amplicons were generated using the nCoV-2019 sequencing protocol v3 (protocols.io), and the integrity of PCR amplicons was checked with a fragment analyzer (Agilent). The library preparation for sequencing was done on Biomek FX (Beckman) with the KAPA hyper prep kit for DNA (Roche). Truncated universal stub adapters were used for ligation, and indexed primers were used during PCR amplification to complete the adapters and to enrich the libraries for adapter-ligated fragments. Samples were checked for quality on a fragment analyzer. Samples were quantified by Illumina Quant qPCR Kit (Kapa Biosystems), pooled evenly by molarity and sequenced on an Illumina NovaSeq6000 150PE S4. Fastq files were further processed to produce consensus sequences and allele frequencies using trimmomatic (v.0.39.2), bowtie2 (v.2.4.4), samtools (v.1.14), iVar (v.1.3.1), bcftools (v.1.14) and perbase (v.0.8.1). Sequences were uploaded to GenBank with accession numbers OM319524 and to SRA at SRR17658563.

### Production of other virus stocks

Viral stocks for MERS-CoV (BEI Resources, catalog no. NR-48813) and HKU5-SARS-CoV-1-S (BEI Resources, catalog no. NR-48814) were generated by inoculating Vero E6 cells with ~0.01 MOI for 3 d to create a p1 stock. The p1 stock was used to inoculate Vero E6 cells and after 3 d, with ~50% CPE, supernatants were harvested, spun (450x*g* for 5 min) and filtered through a 0.45 µm syringe filter. Aliquoted viruses were stored at −80 °C. Plaque assay was used to calculate the titer of infectious virus in the generated stocks. Stocks of PIV3 (PIV3–GFP, ~10^7.4^ TCID_50_ per ml) and RSV (RSV–GFP1, ~10^7^ TCID_50_ per ml) were obtained from Viratree. Stocks were then expanded in T225 flasks of 80% confluent LLC-MK2 or Hep-2 cells, respectively, by inoculating cells with 100 µl of virus stocks. The CPE was monitored daily and flasks were frozen when cells exhibited ~70% CPE, usually ~72–96 h postinfection. Lysates were thawed, collected and cell debris was spun down at 10,000x*g* for 10 min. The clarified virus-containing supernatants were then aliquoted and infectious viral particles were quantified with a GFP^+^ flow limiting-dilution assay using LLC-MK2 cells. HCoV-229E, HCoV-OC43 and influenza virus strain PR8 were obtained from ATCC. Stocks of HCoV-229E, HCoV-OC43 and PR8 were amplified in MRC5, HCT8 and MDCK cells respectively. For each of these viruses, stocks were prepared by infecting a T150 flask of cells with 50 µl of ATCC stock. Flasks were frozen down once 60–70% CPE was observed. Lysates were then thawed, collected and cell debris was spun down at 10,000x*g* for 10 min. The clarified virus-containing supernatants were then aliquoted and infectious viral particles were quantified via TCID_50_.

### Generation of replication competent VSV pseudovirus

Recombinant VSV expressing eGFP and SARS-CoV-2-S (VSVdG-eGFP-CoV-2-S) was generated as previously described^16^. In short, VSVdG-eGFP (Addgene, plasmid #31842) was modified to insert in frame with the deleted VSV-G a codon-optimized SARS-CoV-2-S based on the Wuhan-Hu-1 isolate (GenBank: MN908947.3), which was mutated to remove a putative ER retention domain (K1269A and H1271A). The virus was rescued and passaged in Huh7.5.1 cells until widespread GFP fluorescence and CPEs were observed. Virus was propagated on Vero E6 cells and titrated on Vero E6 cells overexpressing TMPRSS2. Sequencing revealed additional mutations at the C terminus (1274STOP) and a partial mutation at A372T (~50%) in the ectodomain. Similar adaptive mutations were found in a previous published VSVdG-CoV-2-S^72^.

Recombinant VSV expressing eGFP and rabies virus G (VSVdG-eGFP-RABV-G) was generated in a similar manner. Rabies virus (RABV) glycoprotein (G) was amplified from a template containing the G sequence from SAD-B19. This gene was assembled into VSV-eGFP-dG (Addgene, plasmid #31842) inframe with the G coding sequence between MluI and NotI to generate VSV-eGPF-RABV-G. To rescue the VSVdG-RABV-G, 293FT cells (Thermo Fisher Scientific) were co-cultured with Vero E6 cells in a six-well plate. Cells were transfected with pCAGEN-VSV-N (300 ng), pCAGEN-VSV-P (500 ng), pCAGEN-VSV-L (200 ng), pCAGEN-VSV-G (800 ng), pCAGGS-T7 (200 ng) and VSV-eGFP-dG-RABV-G (500 ng) using JetPrime (Polyplus). Media was changed to DMEM + 2% FBS after 1 d, and cells were observed until widespread GFP was observed by day 7. Rescued virus was plaque purified, propagated and titrated on Vero E6 cells. Sequencing confirmed that the sequence matched the SAD-B19 and no mutations were observed.

### Mucin KO mouse experiments

Mice deficient in the three major transmembrane mucins (Muc1, Muc4 and Muc16) were used to confirm a role for transmembrane mucins in SARS-CoV-2 infection in vivo. Mice were generated by breeding individual Muc1-, Muc4- and Muc16-deficient mice (C57BL/6NTac congenic) to form triple transgenic homozygous Muc1/4/16-deficient mice^73–75^. WT control mice were provided by C57Bl/6NTac mice bred in the same colony. C57BL/6NTac mice (15–20 weeks old), of both genders, were used for in vivo experiments. All mice were housed individually in ventilated microisolator cages in a facility maintained at the University of North Carolina at Chapel Hill, on a 12 h day/night cycle. Mice were fed a regular chow diet and given water ad libitum until the defined experimental endpoints. Researchers were not blinded during in vivo experiments. Triple deficient mice and WT mice were treated with mouse-adapted SARS-CoV-2 (10^3^ p.f.u.) according to standard protocols and lungs were harvested at 2 d postinfection and lungs processed according to approved protocols^76^. IHC for SARS-CoV-2 nucleocapsid protein (Invitrogen, catalog no. PA1-41098) was performed on paraffin-embedded 5-μm tissue sections to detect virus followed by 3,3′-diaminobenzidine (DAB) development and hematoxylin II staining (performed by Animal Histopathology & Laboratory Medicine Core at the University of North Carolina). RNA-ISH was performed on paraffin-embedded 5-μm tissue sections using the RNAscope Multiplex Fluorescent Assay v.2 according to the manufacturer’s instructions (Advanced Cell Diagnostics) and described previously^77^. Briefly, tissue sections were deparaffinized with xylene and 100% ethanol twice for 5 min and 1 min, respectively, incubated with hydrogen peroxide for 10 min and in boiling water for 15 min, and then incubated with Protease Plus (Advanced Cell Diagnostics) for 15 min at 40 °C. Slides were hybridized with a custom probe for SARS-CoV-2 Spike gene (RNAScope probe SARS-CoV-2, S gene encoding the spike protein, catalog no. 848561) at 40 °C for 2 h, and signals were amplified according to the manufacturer’s instructions. Stained sections were scanned and digitized using an Olympus VS200 microscope with a 20x 0.8 NA objective. Images were imported into Visiopharm Software (v.2020.09.0.8195) for quantification. Lung tissue, SARS-CoV-2 nucleocapsid protein and SARS-CoV-2 Spike gene were quantified using customized analysis protocol packages to: (1) detect lung tissue using a decision forest classifier, (2) detect the DAB signal of SARS-CoV-2 using the green channel alone and threshold set to remove background staining, and (3) detect the probe signal based on the intensity of the Cy5 signal corresponding to SARS-CoV-2 Spike gene probe. All slides were analyzed under the same conditions. All animal work was approved by the Institutional Animal Care and Use Committee at University of North Carolina at Chapel Hill according to guidelines outlined by the Association for the Assessment and Accreditation of Laboratory Animal Care and the U.S. Department of Agriculture. All infection studies were performed in animal biosafety level 3 (BSL-3) facilities at University of North Carolina at Chapel Hill.

### Statistics and data collection

Biological replicates in this study are defined as distinct experiments conducted with a unique aliquot of virus and a unique passage of indicated cells. Comparisons were made by individual *t*-tests comparing gene-edited cell lines to a corresponding NTG control.

### Reporting summary

Further information on research design is available in the Nature Research Reporting Summary linked to this article.

## Online content

Any methods, additional references, Nature Research reporting summaries, source data, extended data, supplementary information, acknowledgements, peer review information; details of author contributions and competing interests; and statements of data and code availability are available at 10.1038/s41588-022-01131-x.

## Supplementary information

Supplementary Information Textual supplementary note of expanded discussion of findings. Separate references to accompany supplementary note.

Reporting Summary

Supplementary Table 1 T1: LOF-enriched screen analysis. T2: GOF-depleted screen analysis. T3: GOF-enriched screen analysis. T4: Guide Sequences (GOF). T5: Guide Sequences (LOF). T6: Knockout efficiencies of cell lines used in this study. T7: P-values for all studies.

## Source data

Source Data Extended Data Fig. 3 Unprocessed western blots and/or gels.

Source Data Extended Data Fig. 4 Unprocessed western blots and/or gels.

Source Data Extended Data Fig. 6 Unprocessed western blots and/or gels.

Source Data Extended Data Fig. 9 Unprocessed western blots and/or gels.

## Data Availability

All raw data associated with all figures are included in this submission and are also available upon request. Publicly available datasets in NCBI were used for Fig. 3 found at accession numbers: GSE145926 and GSM3660650. Publicly available datasets for Fig. 5 are found at accession numbers: GSE147507 (ref. ^4^), GSE152586 (ref. ^63^), GSE154104 (ref. ^64^) and GSE161200. SARS-CoV-2 stocks have been sequenced and raw sequencing data has been deposited to both NCBI GenBank at accession OM319524 and SRA at SRR17658563. Exact *P* values for all statistical comparisons are provided in Supplementary Table 7. Additional details on methodology are available in the attached supplementary note detailing the following experiments: StcE pretreatment experiments, Fluorescence-based infection tracking experiments, SARS-CoV-2 binding assay, qPCR validation of overexpressing cell lines, qPCR quantification for viral genome copies, flow cytometry, western blot, single-cell RNA meta-analysis of TMPRSS2 + ACE2 + ciliated lung epithelial cells, Bulk RNA-seq analysis of mucin gene expression in response to SARS-CoV-2 infection, RNA-seq analysis for COVID-19 clinical samples, and knockout analysis. Source data are provided with this paper.

## Code availability

No new code was generated for analysis. Datasets were analyzed with standard analysis pipelines as detailed in the Methods section.
