# Supplementary material for: Genome-wide bidirectional CRISPR screens identify mucins as host factors modulating SARS-CoV-2 infection
Source: Nat Genet. 2022 Jul 25;54(8):1078–89. doi: 10.1038/s41588-022-01131-x (PMC9355872; doi:10.1038/s41588-022-01131-x)
Supplement: Supplementary file 1 — Textual supplementary note of expanded discussion of findings. Separate references to accompany supplementary note. [file 41588_2022_1131_MOESM1_ESM.pdf]

nature genetics

SUPPLEMENTARY INFORMATION

https://doi.org/10.1038/s41588-022-01131-x

In the format provided by the authors and unedited.

OPEN

Genome-wide bidirectional CRISPR screens identify mucins as host factors modulating SARS-CoV-2 infection

In the format provided by the authors and unedited

SUPPLEMENTARY NOTE

Once SARS-CoV-2 enters an ACE2-expressing cell, the viral RNA genome is translated and its products have a myriad of effects on the host cell - including rearranging membranes to form a viral replication complex and hijacking the secretory pathway for viral egress [1,2]. During the SARS-CoV-2 lifecycle, cell-intrinsic innate immune responses can recognize viral pathogenassociated molecular patterns (PAMPs) and induce an antiviral cellular state. This typically involves activation of cell death programs, production of proinflammatory cytokines via the nuclear factor kappa-light-chain-enhancer of B cells (NF-κB) signaling pathway, and upregulation of type I interferons (IFN), which regulate expression of numerous interferon-stimulated genes (ISGs) [3,4]. In turn, SARS-CoV-2 has evolved numerous strategies to antagonize or reroute these antiviral programs [1]. Host-factors promoting or antagonizing these processes are diverse.

A strength of bi-directional CRISPR screens is that genes and pathways can be interrogated in both a GOF- and LOF-manner in parallel, possessing potential to capture the complexity of hostpathogen interactions. The emergence of inversely complementary hits from GOF and LOF screens (*e.g.* key pathway activators and inhibitors emerging from opposite screen directions) increases confidence in the importance of a given pathway for SARS-CoV-2 infection. Our screens identified several such pathways including cell cycle, NF-κB signaling, and epigenetic modifiers.

While many DNA viruses such as papillomaviruses, adenoviruses, and polyomaviruses actively promote the cell cycle to increase availability of DNA replication machinery [5], the opposite trend is generally seen for RNA viruses [6]. Several members of the *Coronaviridae* family including SARS-CoV-1 and murine hepatitis virus (MHV) employ diverse strategies for cell cycle arrest, which is hypothesized to benefit the virus via redistribution of cellular resources, avoidance of apoptosis-triggering checkpoints, or maintenance of organelle architecture required for viral replication [6–9]. SARS-CoV-2 likely exploits similar approaches, although the specific mechanisms are still emerging. Previous work has demonstrated SARS-CoV-2 genomic RNA directly interacts with the cell cycle promoter CDK2, which also exhibits decreased phosphorylation during infection [10]. We observed that upregulation of factors promoting the cell cycle such as cyclin E1 and cyclin D1 restrict SARS-CoV-2 infection in Calu-3 cells, while overexpression of cell cycle inhibitors like *CDKN1A* and *CDKN2B* enhance SARS-CoV-2 infection. Although some screen hits may be enriched in pooled survival screens due to their effect on cell proliferation independent of viral infection, we validated *CCNE1* and *CDKN2B* in assays directly measuring viral replication. Supporting this, cell cycle-modulating compounds such as dinaciclib and AZD5438 antagonize SARS-CoV-2 replication [10,11]. Taken together, a growing body of work indicates cell cycle arrest is critical for optimal coronavirus infection [9,12–14].

NF-κB-mediated signaling also emerged from bidirectional functional screening. Some factors, such as NF-κB-induced genes *CD44* and *ICAM1*, emerged as putatively antiviral, based on enrichment in the GOF screen. However, the majority of the NF-κB related hits appeared as proviral, with *CHUK/IKK-α* and *RIPK4* enriched in the LOF screen, and NF-κB pathway activators *IL-17C* and its signaling adapter protein ACT1 (*TRAF31P*2) depleted in the GOF screen. Human coronavirus 229E selectively modulates certain components of the NF-κB signaling pathway for

its replicative benefit in an IKK complex-dependent manner [15]. A proviral role of the NF-κB pathway has also been reported for IAV, as NF-κB inhibitors as well as genetic perturbation were demonstrated to restrict IAV infection *in vitro* and *in vivo* [16–19]. A similar mechanism for a proviral role of proinflammatory cytokines and NF-κB-signaling for SARS-CoV-2 would be plausible. Alternatively, a more indirect role via modulation of cell proliferation or cell death pathways would also be possible.

One of the most prominently enriched proviral clusters from our LOF screens was a set of clathrin adaptor proteins from the AP1 complex, which are involved in vesicle formation and intracellular trafficking. Several AP1 adaptor proteins have also been identified as interactors of SARS-CoV-2 M protein, including one of our top validated hits, AP1G1 [20]. Our VSV-CoV-2-S pseudotype assay suggests that AP1 adaptors promote viral entry. Given this observation, a direct role of the AP1 complex in clathrin-mediated endocytosis of SARS-CoV-2 virions should be investigated. In addition, the AP1 complex may also be involved in other stages of the viral life cycle, including intracellular viral trafficking and egress, or be manipulated by the SARS-CoV-2 M protein to reroute host proteins and facilitate viral infection. A similar mechanism is used by HIV-1, which hijacks the AP1 complex to retain the viral restriction factor BST2 within endosomes and drive its lysosomal degradation [21]. Depletion of AP1 components can also alter cell surface expression of membrane proteins [22,23]. As a result, entry factors like ACE2 and TMPRSS2 may be mislocalized in AP1 LOF cells, resulting in decreased viral entry, given that ACE2 cell surface expression levels are not altered in AP1 LOF cells [24].

Another central class of genes identified in our LOF screen were components involved with cytoskeletal rearrangement (*ROCK1*) and cell-cell junctions (*CDH1*/E-cadherin). Viruses commonly co-opt actin or microtubule components and motor proteins to shuttle viral components around the cell and enable efficient replication [25–27]. Beyond intracellular shuttling, some viruses hijack host cytoskeletal components as well as cell-cell protein complexes for cell-cell spread [10,28–30]. An example of this for SARS-CoV-2 is the observation that SARS-CoV-2 nsp7 and N proteins mediate cytoskeletal rearrangement by signaling through *RhoA* and *CK2*, forming filopodial protrusions from infected cells that have been hypothesized to serve as portals of cell-cell spread [10,28,29]. Furthermore, as ACE2 has been reported to be preferentially localized to the cilia of upper and lower respiratory tract epithelial cells, loss of cytoskeletal components could also affect proper cell surface expression of *ACE2* and potentially other SARS-CoV-2 entry factors, subsequently decreasing viral entry [31]. Further, because cell division involves tight coordination between the cytoskeleton, cell-cell junctions, and nuclear components, some of the identified cell junction and cytoskeleton components may also regulate the cell proliferative state, potentially connecting these two pathways.

Our GOF screen identified several G-protein coupled receptors involved in smell and taste signaling as SARS-CoV-2 restriction factors. A key protein interaction network enriched in the GOF screen include the olfactory receptors OR8A1, OR2T33, and OR51S1, the G protein gamma subunit GNG13 that plays a key role in taste transduction, and the GPCR kinase GRK3. Respiratory epithelium and neuroepithelium of the human nasal cavity robustly express *ACE2* and *TMPRSS2*, and transcripts encoding olfactory receptors and *GNG13* are downregulated in human and hamster olfactory epithelium following SARS-CoV-2 infection [32–34]. The specific mechanism

of an antiviral role for olfactory receptors, and their potential connections to COVID-19 symptoms such as anosmia, will warrant further investigation. However, many sensory receptors are expressed in diverse tissues and have functions beyond their canonical role in olfactory epithelium sensory neurons. Taste receptors in airway epithelia can play a direct protective role, sensing inhalation of noxious chemicals as well as metabolic products of pathogens. By activating calcium signaling and other second messenger pathways, they then increase cilia beat frequency to drive mucociliary clearance [35,36]. Furthermore, whole exome studies of infants infected with respiratory syncytial virus (RSV) identified genetic variants in *OR8U1* and *OR8U8*, suggesting that loss of some olfactory receptors could be involved in increased host susceptibility to RSV infection [37]. Mutations in our antiviral screen hit *GRK3* have also been found to be associated with immunodeficiency disorders [38]. Because we did not identify olfactory or gustatory components in the LOF screen, which may not be expressed in lung epithelial cells such as Calu-3 lines, GOF screens highlight the potential to find novel functional pathways via ectopic overexpression.

We also identified membrane-tethered mucins forming a prominent interaction cluster of SARSCoV-2 restriction factors. Mucins are a family of densely O-glycosylated proteins that are the primary constituent of mucus lining epithelial cell barriers in the lungs and gut [39], protecting the respiratory tract from environmental insults such as microbial infection [40,41]. Mucins are highly expressed by epithelial cells [42] and are separated into two major classes. Secreted, gel-forming mucins form the mucus layer (such as *MUC5AC* and *MUC5B*), while transmembrane mucins (such as *MUC1*, *MUC4*, *MUC13*, *MUC21*) are anchored to the apical side of epithelial cells [43,44]. Viral infections, including by SARS-CoV-2, have been shown to upregulate mucin expression in primary lung epithelial cells in a type I IFN-dependent manner, suggesting potential protective roles for mucins [45–47].

Our investigation demonstrates that membrane-tethered mucins serve as SARS-CoV-2 restriction factors in Calu-3 immortalized human lung epithelial cells, primary human epithelial cells, and mice, inhibiting infection at the stage of virus binding to cells **(Figure 5-7)**. This is supported by quantitative trait locus (QTL) and *in vivo* studies suggesting that membrane-tethered *MUC4* could serve a protective role in mice against SARS-CoV-1 and chikungunya virus infection [48]. Membrane-anchored mucins generally act as pan-viral restriction factors, and are capable of inhibiting HKU5-SARS-CoV-1-S, MERS-CoV, 229E, PIV3, and IAV in addition to SARS-CoV2. Intriguingly, we find that some viruses including RSV appear to escape inhibition by membraneanchored mucins, suggesting that virus-mucin interactions may be at the forefront of a molecular arms race between respiratory viruses and their hosts.

In the context of SARS-CoV-2, mucin studies have largely focused on their potentially detrimental functions during the later stages of COVID-19. Overproduction and accumulation of secreted mucins in the lungs can lead to airway obstruction, thereby reducing airflow, exacerbating lung disease, and potentially promoting ARDS [49–51]. It has therefore been proposed to antagonize mucin expression as a therapeutic strategy [46,52,53], and compounds that inhibit MUC1 are now being tested in clinical trials for hospitalized adults with COVID-19 [54,55].

Given our findings of a protective role for membrane-anchored mucins against SARS-CoV-2, the development of therapeutics broadly antagonizing mucin expression should be pursued with

caution. Structural variant studies of COVID-19 cases identified *MUC4* loss-of-function to be associated with increased disease severity [56,57]. Consistent with this, looking at individual human lung epithelial cells from patient BALF, we find that cells containing SARS-CoV2 express lower levels of *MUC1*, supporting a viral restrictive role for membrane-tethered mucins in humans. In light of these data, a more selective strategy promoting membrane-tethered mucins or reducing the secretion of gel-forming mucins may be more beneficial. In line with this, the sole secreted mucin (*MUC5AC*) identified in our screens was depleted in the GOF study and confirmed to promote SARS-CoV-2 infection at the step of entry in Calu-3 cells, suggesting that expression of gelforming mucins *in vivo* may promote viral infection. Taken together, the levels of distinct mucins in the lungs of infected patients may determine the balance between the protective benefit of these surface glycoproteins and the pathological detriment in gas exchange.

Future work is required to determine the exact mechanism of membrane-anchored mucins in restricting SARS-CoV-2 entry. Our study suggests a role of membrane anchored mucins in restricting binding of SARS-CoV2 to the cell surface (**Figure 7f**) and therefore its host receptors - likely through steric hindrance via a denser glycocalyx (**Extended Figure b-d)**. In contrast, IAV interacts with sialic acid residues on host mucins, which can actively trap viral particles and inhibit viral entry [45,58–61]. Defining the molecular interactions between SARS-CoV-2 virions and mucins, and their consequences during SARS-CoV-2 infection *in vivo*, will clarify the potentially bimodal effect of mucins on COVID-19 severity.

Supplementary Notes References

1. Hartenian, E. *et al.* The molecular virology of coronaviruses. *J. Biol. Chem.* **295**, 12910–12934 (2020).
2. V'kovski, P., Kratzel, A., Steiner, S., Stalder, H. & Thiel, V. Coronavirus biology and replication: implications for SARS-CoV-2. *Nat. Rev. Microbiol.* **19**, 155–170 (2020).
3. Katze, M. G., He, Y. & Gale, M., Jr. Viruses and interferon: a fight for supremacy. *Nat. Rev. Immunol.* **2**, 675–687 (2002).
4. Yang, H., Lyu, Y. & Hou, F. SARS-CoV-2 infection and the antiviral innate immune response. *J. Mol. Cell Biol.* **12**, 963–967 (2021).
5. Bagga, S. & Bouchard, M. J. Cell Cycle Regulation During Viral Infection. in *Cell Cycle Control: Mechanisms and Protocols* (eds. Noguchi, E. & Gadaleta, M. C.) 165–227 (Springer New York, 2014).
6. Fan, Y., Sanyal, S. & Bruzzone, R. Breaking bad: How viruses subvert the cell cycle. *Front.*

Cell. Infect. Microbiol. **8**, 396 (2018).

7. Dyer, M. D., Murali, T. M. & Sobral, B. W. The Landscape of Human Proteins Interacting with Viruses and Other Pathogens. *PLoS Pathog.* **4**, e32 (2008).

8. Su, M. *et al.* A mini-review on cell cycle regulation of Coronavirus infection. *Front. Vet. Sci.* **7**, 586826 (2020).

9. Yuan, X. *et al.* SARS coronavirus 7a protein blocks cell cycle progression at G0/G1 phase via the cyclin D3/pRb pathway. *Virology* **346**, 74–85 (2006).

10. Bouhaddou, M. *et al.* The Global Phosphorylation Landscape of SARS-CoV-2 Infection. *Cell* **182**, 685–712.e19 (2020).

11. Biering, S. B. *et al.* Screening a Library of FDA-Approved and Bioactive Compounds for Antiviral Activity against SARS-CoV-2. *ACS Infect Dis* **7**, 2337–2351 (2021).

12. Cawood, R., Harrison, S. M., Dove, B. K., Reed, M. L. & Hiscox, J. A. Cell cycle dependent nucleolar localization of the coronavirus nucleocapsid protein. *Cell Cycle* **6**, 863–867 (2007).

13. Mizutani, T. *et al.* Inhibition of cell proliferation by SARS-CoV infection in Vero E6 cells. *FEMS Immunol. Med. Microbiol.* **46**, 236–243 (2006).

14. Yuan, X. *et al.* G1 phase cell cycle arrest induced by SARS-CoV 3a protein via the cyclin D3/pRb pathway. *Am. J. Respir. Cell Mol. Biol.* **37**, 9–19 (2007).

15. Poppe, M. *et al.* The NF-κB-dependent and -independent transcriptome and chromatin landscapes of human coronavirus 229E-infected cells. *PLoS Pathog.* **13**, e1006286 (2017).

16. Ehrhardt, C. *et al.* The NF-κB inhibitor SC75741 efficiently blocks influenza virus propagation and confers a high barrier for development of viral resistance. *Cell. Microbiol.* **15**, 1198–1211 (2013).

17. Kumar, N., Xin, Z.-T., Liang, Y., Ly, H. & Liang, Y. NF-kappaB signaling differentially regulates influenza virus RNA synthesis. *J. Virol.* **82**, 9880–9889 (2008).
18. Pinto, R. *et al.* Inhibition of influenza virus-induced NF-kappaB and Raf/MEK/ERK activation can reduce both virus titers and cytokine expression simultaneously in vitro and in vivo. *Antiviral Res.* **92**, 45–56 (2011).
19. Schmitz, M. L., Kracht, M. & Saul, V. V. The intricate interplay between RNA viruses and NF-κB. *Biochim. Biophys. Acta* **1843**, 2754–2764 (2014).
20. Chen, Z. *et al.* Interactomes of SARS-CoV-2 and human coronaviruses reveal host factors potentially affecting pathogenesis. *EMBO J.* **40**, e107776 (2021).
21. Jia, X. *et al.* Structural basis of HIV-1 Vpu-mediated BST2 antagonism via hijacking of the clathrin adaptor protein complex 1. *Elife* **3**, e02362 (2014).
22. Castillon, G. A., Burriat-Couleru, P., Abegg, D., Criado Santos, N. & Watanabe, R. Clathrin and AP1 are required for apical sorting of glycosyl phosphatidyl inositol-anchored proteins in biosynthetic and recycling routes in Madin-Darby canine kidney cells. *Traffic* **19**, 215–228 (2018).
23. Takahashi, D. *et al.* The epithelia-specific membrane trafficking factor AP-1B controls gut immune homeostasis in mice. *Gastroenterology* **141**, 621–632 (2011).
24. Rebendenne, A. *et al.* Bidirectional genome-wide CRISPR screens reveal host factors regulating SARS-CoV-2, MERS-CoV and seasonal coronaviruses. *bioRxiv* 2021.05.19.444823 (2021) doi:10.1101/2021.05.19.444823.
25. Arons, M. M. *et al.* Presymptomatic SARS-CoV-2 Infections and Transmission in a Skilled Nursing Facility. *N. Engl. J. Med.* **382**, 2081–2090 (2020).

26. Taylor, M. P., Koyuncu, O. O. & Enquist, L. W. Subversion of the actin cytoskeleton during viral infection. *Nat. Rev. Microbiol.* **9**, 427–439 (2011).

27. Wen, Z., Zhang, Y., Lin, Z., Shi, K. & Jiu, Y. Cytoskeleton—a crucial key in host cell for coronavirus infection. *J. Mol. Cell Biol.* **12**, 968–979 (2020).

28. Bergelson, J. M. Intercellular junctional proteins as receptors and barriers to virus infection and spread. *Cell Host Microbe* **5**, 517–521 (2009).

29. Gordon, D. E. *et al.* A SARS-CoV-2 protein interaction map reveals targets for drug repurposing. *Nature* **583**, 459–468 (2020).

30. Mothes, W., Sherer, N. M., Jin, J. & Zhong, P. Virus Cell-to-Cell Transmission. *J. Virol.* **84**, 8360–8368 (2010).

31. Lee, I. T. *et al.* ACE2 localizes to the respiratory cilia and is not increased by ACE inhibitors or ARBs. *Nat. Commun.* **11**, 5453 (2020).

32. Brann, D. H. *et al.* Non-neuronal expression of SARS-CoV-2 entry genes in the olfactory system suggests mechanisms underlying COVID-19-associated anosmia. *Sci Adv* **6**, (2020).

33. Fodoulian, L. *et al.* SARS-CoV-2 Receptors and Entry Genes Are Expressed in the Human Olfactory Neuroepithelium and Brain. *iScience* **23**, 101839 (2020).

34. Zazhytska, M. *et al.* Disruption of nuclear architecture as a cause of COVID-19 induced anosmia. *bioRxiv* 2021.02.09.430314 (2021) doi:10.1101/2021.02.09.430314.

35. Lee, R. J. *et al.* T2R38 taste receptor polymorphisms underlie susceptibility to upper respiratory infection. *J. Clin. Invest.* **122**, 4145–4159 (2012).

36. Shah, A. S., Ben-Shahar, Y., Moninger, T. O., Kline, J. N. & Welsh, M. J. Motile cilia of human airway epithelia are chemosensory. *Science* **325**, 1131–1134 (2009).

37. Salas, A. *et al.* Whole Exome Sequencing reveals new candidate genes in host genomic susceptibility to Respiratory Syncytial Virus Disease. *Sci. Rep.* **7**, 15888 (2017).

38. Balabanian, K. *et al.* Leukocyte analysis from WHIM syndrome patients reveals a pivotal role for GRK3 in CXCR4 signaling. *J. Clin. Invest.* **118**, 1074–1084 (2008).

39. Wagner, C. E., Wheeler, K. M. & Ribbeck, K. Mucins and Their Role in Shaping the Functions of Mucus Barriers. *Annu. Rev. Cell Dev. Biol.* **34**, 189–215 (2018).

40. Button, B. *et al.* A periciliary brush promotes the lung health by separating the mucus layer from airway epithelia. *Science* **337**, 937–941 (2012).

41. Kesimer, M. *et al.* Molecular organization of the mucins and glycocalyx underlying mucus transport over mucosal surfaces of the airways. *Mucosal Immunol.* **6**, 379–392 (2013).

42. Ma, S., Meng, Z., Chen, R. & Guan, K.-L. The Hippo Pathway: Biology and Pathophysiology. *Annu. Rev. Biochem.* **88**, 577–604 (2019).

43. Carson, D. D. The Cytoplasmic Tail of MUC1: A Very Busy Place. *Sci. Signal.* **1**, e35–pe35 (2008).

44. Hattrup, C. L. & Gendler, S. J. Structure and function of the cell surface (tethered) mucins. *Annu. Rev. Physiol.* **70**, 431–457 (2008).

45. Iverson, E. *et al.* Membrane-Tethered Mucin 1 is Stimulated by Interferon in Multiple Cell Types and Antagonizes Influenza A Virus Infection in Human Airway Epithelium. *bioRxiv* 2021.03.11.434997 (2021) doi:10.1101/2021.03.11.434997.

46. Liu, Y. *et al.* Mucus production stimulated by IFN-AhR signaling triggers hypoxia of COVID-19. *Cell Res.* **30**, 1078–1087 (2020).

47. Lu, W. *et al.* Elevated MUC1 and MUC5AC mucin protein levels in airway mucus of critical ill COVID-19 patients. *J. Med. Virol.* **93**, 582–584 (2021).

48. Plante, J. A. *et al.* Mucin 4 Protects Female Mice from Coronavirus Pathogenesis. *Cold Spring Harbor Laboratory* 2020.02.19.957118 (2020) doi:10.1101/2020.02.19.957118.

49. He, J. *et al.* Single-cell analysis reveals bronchoalveolar epithelial dysfunction in COVID-19 patients. *Protein & cell* vol. 11 680–687 (2020).

50. Nakashima, T. *et al.* Circulating KL-6/MUC1 as an independent predictor for disseminated intravascular coagulation in acute respiratory distress syndrome. *J. Intern. Med.* **263**, 432–439 (2008).

51. Vestbo, J. Epidemiological studies in mucus hypersecretion. *Novartis Found. Symp.* **248**, 3–12; discussion 12–9, 277–82 (2002).

52. Guan, W.-J., Chen, R.-C. & Zhong, N.-S. Strategies for the prevention and management of coronavirus disease 2019. *Eur. Respir. J.* **55**, (2020).

53. Kost-Alimova, M. *et al.* A High-Content Screen for Mucin-1-Reducing Compounds Identifies Fostamatinib as a Candidate for Rapid Repurposing for Acute Lung Injury. *Cell Reports Medicine* **1**, 100137 (2020).

54. Strich, J. R. Fostamatinib for Hospitalized Adults With COVID-19. (2020).

55. Tabassum, N., Zhang, H. & Stebbing, J. Repurposing Fostamatinib to Combat SARS-CoV-2-Induced Acute Lung Injury. *Cell Rep Med* **1**, 100145 (2020).

56. Reay, W. R., Geaghan, M. P., Cairns, M. J. & 23andMe Research Team. Genome-wide meta-analysis of pneumonia suggests a role for mucin biology and provides novel drug repurposing opportunities. *bioRxiv* (2021) doi:10.1101/2021.01.24.21250424.

57. Sahajpal, N. S. *et al.* Host genome analysis of structural variations by Optical Genome Mapping provides clinically valuable insights into genes implicated in critical immune, viral infection, and viral replication pathways in patients with severe COVID-19. *medRxiv*

2021.01.05.21249190 (2021).

58. Cohen, M. *et al.* Influenza A penetrates host mucus by cleaving sialic acids with neuraminidase. *Virol. J.* **10**, 321 (2013).

59. Delaveris, C. S., Webster, E. R., Banik, S. M., Boxer, S. G. & Bertozzi, C. R. Membranetethered mucin-like polypeptides sterically inhibit binding and slow fusion kinetics of influenza A virus. *Proc. Natl. Acad. Sci. U. S. A.* **117**, 12643–12650 (2020).

60. Ehre, C. *et al.* Overexpressing mouse model demonstrates the protective role of Muc5ac in the lungs. *Proc. Natl. Acad. Sci. U. S. A.* **109**, 16528–16533 (2012).

61. McAuley, J. L. *et al.* The cell surface mucin MUC1 limits the severity of influenza A virus infection. *Mucosal Immunol.* **10**, 1581–1593 (2017).

Supplementary Methods

StcE pretreatment experiments

Calu-3 cells were plated in clear 96-well plates for infection at 70% confluence the next day. Media was removed from cells and 100uL of RPMI+ 5µg/mL of StcE was added to cells for 2 hours. Virus was then diluted in 100uL of RPMI+10% FBS and added to cells for 24 hours before washing and lysing for a viral genome copy qPCR assay (outlined below). Infections were conducted at the following MOIs: PIV3 (MOI 0.0002), RSV (MOI 0.003), HCoV-229E (MOI 0.05), HCoV-OC43 (MOI 0.05), Influenza A virus PR8 (MOI 0.005) in 200 µL of RPMI+ 5% FBS. The same procedure as above was used for infecting Calu-3 cells with HKU5-SARS-CoV-1-S, SARS-CoV-2 or MERS-CoV at a MOI of 0.1. 24 hours post infection the plate was harvested for plaque assays to quantify viral infection.

Fluorescence-based infection tracking experiments

Calu-3 cells overexpressing CRISPR activator components and *MUC4*- and NTG-targeting sgRNAs were seeded into 96-well plates, targeting 30% density for the next day. After allowing for attachment, media was changed to RPMI+2% FBS. 22 hours later, StcE mucinase diluted in RPMI+2% FBS was spiked into mucinase-treated wells to achieve a final concentration of 20nM mucinase. Vehicle conditions received RPMI+2% FBS to maintain equal well volumes. 2 hours later, the pseudovirus VSVdG-eGFP-CoV-2-S and control VSVdG-eGFP-RABV-G were added to achieve MOI 0.1, then centrifuged at 900xg at 30°C for 60 minutes. After spinfection, individual

wells were imaged over time using an Incucyte System (Sartorius) in a 37°C incubator at 5% CO_2. Images were taken of each well at 4x magnification, and infection was tracked by GFP. The number of GFP-positive foci per well was calculated using Incucyte Analysis Software.

SARS-CoV-2 binding assay

To investigate levels of SARS-CoV-2 binding to Calu-3 cells, virus was added to cells on ice and allowed to incubate for 1 hour. Cells then underwent four washes with ice-cold 1xPBS to wash away the unbound virus. Cell lysates were then collected for quantitation of viral RNA using RT-qPCR.

qPCR validation of overexpressing cell lines

Cells were cultured in 96-well plates until >70% confluency, and cDNA was acquired with direct lysis to reverse transcription protocol as described in [1]. Taqman qRT-PCR was performed with the following probes: *TEAD3* (Hs00243231_m1), *ZNF275* (Hs01010271_m1) *CCNE1* (Hs01026536_m1), *SPDEF* (Hs00171942_m1), *JDP2* (Hs00185689_m1), *TAF7L* (Hs00227589_m1), *IRF5* (Hs00158114_m1), *MUC1* (Hs00159357_m1), *MUC4* (Hs00366414_m1), *MUC13* (Hs00217230_m1), *MUC21* (Hs01379324_g1), *CD44* (Hs01075861_m1), *MUC5AC* (Hs01365616_m1), *MUC5B* (Hs00861588_m1), *GAPDH* (Hs02786624_g1) was used as an endogenous control gene.

qPCR quantification for viral genome copies

Cells were cultured in 96-well plates until >70% confluency. Then infected with virus for 24 hours before cDNA was acquired with direct lysis to reverse transcription protocol as described in [1]. Taqman qRT-PCR was performed with the following probes: RSV (A antigen, Vi99990014_po), PIV3 (Vi06439670_s1), 229E (Vi06439671_s1), OC43 (Vi06439646_s1), PR8 (pan-flu, VI99990011_PO), SARS-CoV-2 was a custom designed probe targeting the N-gene consisting of SARS-CoV-2 forward primer (AAATTTTGGGGACCAGGAAC), SARS-CoV-2 reverse primer (TGGCACCTGTGTAGGTCAAC) and N-gene Probe (ATGTCGCGCATTGGCATGGA), RNAse-P was used as the endogenous control gene, consisting of a forward primer (IDT, 10006836), reverse primer (IDT,10006837) and probe(IDT, 2930664083).

Flow Cytometry

Calu-3 cells were uplifted and treated with 5ug/mL StcE at 37°C for an hour. Cells were then spun down, resuspended in block buffer for 10 minutes at room temperature (FBS stain buffer, BD Pharmigen) supplemented with 2% FBS and 0.1% BSA (Thermofisher). Cells were then incubated in a 1:500 dilution of PE-conjugated anti-CD44 antibody (Biolegend, 338807) in stain buffer for 30 minutes at room temperature. After 3 washes with stain buffer, flow cytometry on an Attune NxT (Thermofisher) was used to measure PE signal followed by analysis with FlowJo software.

Western Blot

For StcE-treated conditions, Calu-3 cells were uplifted and treated with 5 µg/mL StcE at 37°C for an hour, before lysis with RIPA buffer (Thermo Fisher) for 30 minutes on ice. BCA assay (Thermo Fisher) was used to quantify and normalize protein levels prior to gel loading. 10 µg of sample was mixed with 2x Reducing Laemmli Loading buffer and boiled for 5 minutes and loaded into a Novex™ WedgeWell™ 10%, Tris-Glycine gel. The gel was run at 100V for 70 minutes. Proteins were then transferred onto a 0.2um Nitrocellulose membrane 100V for 90 minutes. The membrane was blocked with Intercept Blocking buffer (LiCor) for an hour at room temperature. The primary antibody was added at a 1:1000 dilution in Tris-buffered Saline (TBST) for an hour at room temperature. Following 3 thorough rinses with TBST, the membrane was incubated with IR800-conjugated anti-mouse secondary antibody (LiCor, 926-32210), or alternatively with an anti-rabbit secondary antibody (LiCor, 926-32211) for an hour at room temperature. After 3 thorough rinses with TBST, the western blot was imaged on an Odyssey Clx with CD44 illuminated in the 800nm channel.

Gene targets were probed with the following primary antibodies: CD44 with primary anti-CD44 antibody (Biolegend, 338807). MUC1 with primary anti-MUC1 antibody (Sigma-Aldrich, 05-652), MUC4 with primary anti-MUC4 antibody (Thermofisher, 35-4900), MUC5AC with primary anti-MUC5AC (Abcam, ab198294), ROCK1 (Proteintech, 21850-1-AP), ACE2 with primary anti-ACE2 antibody (Proteintech, 2115-1-AP). β-Actin was probed as a loading control using an HRP-conjugated primary anti-B-actin antibody (Santa Cruz Biotechnologies, sc-47778 HRP) at a 1:1000 dilution. After 3 thorough washes, an ECL western blot substrate kit (abcam, ab65623) was used to detect HRP. The blots were imaged for chemiluminescence on a Biorad ChemiDoc XRS System, followed by imaging in brightfield to capture the ladder in the same frame as loading controls.

Single-cell RNA meta-analysis of TMPRSS2+ACE2+ ciliated lung epithelial cells

AUC values for transcripts for ciliated human lung epithelial cells co-expressing ACE2 and TMPRSS2 were obtained from published supplementary data from [2]. AUC values for the top 100 hits based on MAGeCK rank for each screen that were present in the dataset were retrieved and sorted high to low for each screen in the screen comparison.

Bulk RNA-seq analysis of mucin gene expression in response to SARS-CoV-2 infection

We identified previously reported RNA-seq datasets of diverse cells and tissues after SARS-CoV-2 infection in order to examine differential mucin gene expression. We derived Calu3- cell differential gene expression data and post-mortem human lung data from GSE147507 [3], human alveolar organoid data and human lung graft data from GSE152586 [4], mouse lung data from GSE154104 [5], and hamster trachea and hamster lung data from GSE161200 [6]. $\log_2$fold-change of transcript expression levels upon SARS-CoV-2 infection and false discovery rates (FDR) for the mucin genes were identified from each corresponding manuscript's reported values. Missing values indicate no expression data was reported for that gene in the given dataset.

RNA-seq analysis for COVID-19 clinical samples

Filtered feature-barcode matrices for 13 BALF samples (4 healthy controls, 9 individuals with COVID-19 were obtained from GSE145926 and GSM3660650 (NCBI). The standard pre-processing workflow in Seurat was followed. Samples were analyzed and further filtered down using quality control metrics to remove batch effects. After removing unwanted cells, each sample was normalized using 'LogNormalize' and integrated into one assay using Seurat v4. Linear transformation was applied to the data using 'ScaleData' prior to dimensional reduction. The top 2,000 variable genes were identified using the 'FindVariableFeatures' method and were used to perform PCA. UMAP was then performed using the top 50 principal components. Seurat uses a graph-based approach to cluster cells which was applied here using the 'FindNeighbors' and 'FindClusters' functions. Once each cell was assigned to a cluster, genetic markers KRT18, KRT19 and TPPP3 were used to identify epithelial cell clusters. The integrated assay was then subsetted to only include epithelial cells. The cells were regrouped based on the sample donor's disease state. Gene expression data was extracted from the epithelial cell subset using 'GetAssayData' on its RNA assay. Finally, differentially expressed genes were identified between the healthy control cells and COVID19-infected cells using the 'FindMarkers' method. We calculated differential gene expression using the scDD R package [7] with the hyperparameters a0 = 0.01, b0 = 0.01, mu0 = 0, s0 = 0.01, and alpha = 0.01.

Epithelial cell clusters were manually annotated with epithelial cell types using marker genes *FOXJ1* (ciliated), *C20orf85* (ciliated), *SOX4* (epithelial progenitor), *DDIT3* (epithelial progenitor), *ATF3* (epithelial progenitor), *MUC5B* (goblet/club), and *PTPRC* (immune). To identify single cells infected by SARS-CoV-2, we used the previously described Viral-Track method [8]. Original FASTQ files were downloaded from GEO. We created a STAR reference genome containing the human genome (Ensembl) and 10,647 viral genomes, including that of SARS-CoV-2 (http://www.virusite.org/). We used UMI-tools (https://github.com/CGATOxford/UMI-tools) to move the 10X barcode and UMI from the R1 FASTQ file to the header of the R2 FASTQ file using the `umi_tools whitelist` and `umi_tools extract` command line utilities. Because the default umi_tools cell calling parameters were more stringent than those of cellranger and Seurat, we used option `--set-cell-number=20000` to retain more cells. We used the Viral-Track pipeline (https://github.com/PierreBSC/Viral-Track) to map the FASTQ files to the merged reference genome and filter known artifacts from the mapping results [8]. Finally, we demultiplexed the viral read counts per sample into viral read counts per cell barcode, loaded the viral read counts into the Seurat metadata, and classified any cell with at least one viral unique molecular identifier (UMI) detected as a viral RNA-positive cell (vRNA+). Among *MUC1, MUC4, MUC13, MUC21,* and *CD44*, only *MUC1* and *MUC4* had median expression greater than 0 in epithelial progenitors for at least one patient disease status group, and thus were included in the analysis. Differences in gene expression between patient disease status groups were tested for significance using the Mann-Whitney U test.

Knockout Analysis

PCR primers specific for the edited locus with overhangs compatible with TrueSeq v2 adapters were designed using Primer-BLAST [9] with parameters for products to be in the range of 150 – 300 base pairs and to minimize off target amplification. Genomic DNA of knockout cell lines were extracted using QuickExtract (Lucigen) per the manufacturer's protocol. Extracted gDNA was

amplified using KAPA HiFi HotStart ReadyMix (Roche) according to the manufacturer's protocol. Bands at the correct size were then gel extracted using the Monarch DNA Gel Extraction Kit (NEB). Purified PCR products were then amplified again using TrueSeq v2 adapters with unique barcodes using the same PCR protocol as defined by the KAPA HiFi HotStart ReadyMix kit, with fewer cycles. Bands at the correct size were gel extracted and purified using Ampure XP PCR Purification beads (Beckman) per the manufacturer's protocol. Samples were pooled by molar concentration and sequenced using MiSeq Reagent Kits v2 (300 cycle) (Illumina). Reads were then analyzed with CRISPResso2 [10] for quantification of indel events. Reported efficiency was the percentage of modified reads for knockout and NTG lines.

1. Joung, J. *et al.* Genome-scale CRISPR-Cas9 knockout and transcriptional activation screening. *Nat. Protoc.* **12**, 828–863 (2017).
2. Muus, C. *et al.* Single-cell meta-analysis of SARS-CoV-2 entry genes across tissues and demographics. *Nat. Med.* **27**, 546–559 (2021).
3. Blanco-Melo, D. *et al.* Imbalanced Host Response to SARS-CoV-2 Drives Development of COVID-19. *Cell* **181**, 1036–1045.e9 (2020).
4. Katsura, H. *et al.* Human Lung Stem Cell-Based Alveolospheres Provide Insights into SARS-CoV-2-Mediated Interferon Responses and Pneumocyte Dysfunction. *Cell Stem Cell* **27**, 890–904.e8 (2020).
5. Winkler, E. S. *et al.* SARS-CoV-2 infection of human ACE2-transgenic mice causes severe lung inflammation and impaired function. *Nat. Immunol.* **21**, 1327–1335 (2020).
6. Hoagland, D. A. *et al.* Leveraging the antiviral type I interferon system as a first line of defense against SARS-CoV-2 pathogenicity. *Immunity* **54**, 557–570.e5 (2021).
7. Korthauer, K. D. *et al.* A statistical approach for identifying differential distributions in single-cell RNA-seq experiments. *Genome Biol.* **17**, 222 (2016).
8. Bost, P. *et al.* Host-Viral Infection Maps Reveal Signatures of Severe COVID-19 Patients. *Cell* **181**, 1475–1488.e12 (2020).

9. Ye, J. *et al.* Primer-BLAST: a tool to design target-specific primers for polymerase chain reaction. *BMC Bioinformatics* **13**, 134 (2012).

10. Clement, K. *et al.* CRISPResso2 provides accurate and rapid genome editing sequence analysis. *Nat. Biotechnol.* **37**, 224–226 (2019).
