# Supplementary material for: Genome-wide bidirectional CRISPR screens identify mucins as host factors modulating SARS-CoV-2 infection
Source: Nat Genet. 2022 Jul 25;54(8):1078–89. doi: 10.1038/s41588-022-01131-x (PMC9355872; doi:10.1038/s41588-022-01131-x)
Supplement: Supplementary file 2 — Reporting Summary [file 41588_2022_1131_MOESM2_ESM.pdf]

nature research

Corresponding author(s): Patrick D. Hsu, Silvana Konermann, Eva Harris

Last updated by author(s): April 11, 2022

# Reporting Summary

Nature Research wishes to improve the reproducibility of the work that we publish. This form provides structure for consistency and transparency in reporting. For further information on Nature Research policies, see our Editorial Policies and the Editorial Policy Checklist.

## Statistics

For all statistical analyses, confirm that the following items are present in the figure legend, table legend, main text, or Methods section.

| n/a | Confirmed | |
|---|---|---|
| ☐ | ☒ | The exact sample size (*n*) for each experimental group/condition, given as a discrete number and unit of measurement |
| ☐ | ☒ | A statement on whether measurements were taken from distinct samples or whether the same sample was measured repeatedly |
| ☐ | ☒ | The statistical test(s) used AND whether they are one- or two-sided<br>*Only common tests should be described solely by name; describe more complex techniques in the Methods section.* |
| ☐ | ☒ | A description of all covariates tested |
| ☐ | ☒ | A description of any assumptions or corrections, such as tests of normality and adjustment for multiple comparisons |
| ☐ | ☒ | A full description of the statistical parameters including central tendency (e.g. means) or other basic estimates (e.g. regression coefficient) AND variation (e.g. standard deviation) or associated estimates of uncertainty (e.g. confidence intervals) |
| ☐ | ☒ | For null hypothesis testing, the test statistic (e.g. *F*, *t*, *r*) with confidence intervals, effect sizes, degrees of freedom and *P* value noted<br>*Give P values as exact values whenever suitable.* |
| ☒ | ☐ | For Bayesian analysis, information on the choice of priors and Markov chain Monte Carlo settings |
| ☒ | ☐ | For hierarchical and complex designs, identification of the appropriate level for tests and full reporting of outcomes |
| ☒ | ☐ | Estimates of effect sizes (e.g. Cohen's *d*, Pearson's *r*), indicating how they were calculated |

*Our web collection on statistics for biologists contains articles on many of the points above.*

## Software and code

Policy information about availability of computer code

| | |
|---|---|
| Data collection | No software was used for data collection. |
| Data analysis | n/a |

For manuscripts utilizing custom algorithms or software that are central to the research but not yet described in published literature, software must be made available to editors and reviewers. We strongly encourage code deposition in a community repository (e.g. GitHub). See the Nature Research guidelines for submitting code & software for further information.

## Data

Policy information about availability of data

All manuscripts must include a data availability statement. This statement should provide the following information, where applicable:

- Accession codes, unique identifiers, or web links for publicly available datasets
- A list of figures that have associated raw data
- A description of any restrictions on data availability

All raw data associated with all figures are included in this submission and are also available upon request. Publicly available data sets were used for Figure 3 found at accession numbers: GSE145926 and GSM3660650. Publicly available data sets for Figure 5 are found at accession numbers: GSE147507 (Blanco-Melo et al., 2020), GSE152586 (Katsura et al., 2020), GSE154104 (Winkler et al., 2020), and GSE161200. SARS-CoV-2 stocks have been sequenced and raw sequencing data has been deposited to both NCBI GenBank at accession OM319524 and SRA at SRR17658563.

# Field-specific reporting

Please select the one below that is the best fit for your research. If you are not sure, read the appropriate sections before making your selection.

☒ Life sciences ☐ Behavioural & social sciences ☐ Ecological, evolutionary & environmental sciences

For a reference copy of the document with all sections, see nature.com/documents/nr-reporting-summary-flat.pdf

# Life sciences study design

All studies must disclose on these points even when the disclosure is negative.

| | |
|---|---|
| Sample size | No sample size calculation was conducted. We selected samples sizes of sufficient size to ensure reproducibility of our findings as well as large enough to perform statistical analyses (at least n=3 unless stated otherwise). |
| Data exclusions | No data were excluded from this study. We pre-established our exclusion criteria as a rejection of a dataset if either our positive controls or negative controls failed. |
| Replication | We repeated experiments (including genome-wide screening) at least three times or the number indicated in the figures legends. All attempts to repeat findings were succesful. |
| Randomization | Experiments were not randomized. Data variability was controlled through the inclusion of multiple biological replicates, inclusion of multiple technical replicates within an experiment, and utilization of distinct guide RNAs targeting a single gene in multiple cell lines. |
| Blinding | Experiments involving cell culture were set up by one investigator and conducted by another in a blind fashion. For other experiments, researchers were not blinded. |

# Reporting for specific materials, systems and methods

We require information from authors about some types of materials, experimental systems and methods used in many studies. Here, indicate whether each material, system or method listed is relevant to your study. If you are not sure if a list item applies to your research, read the appropriate section before selecting a response.

## Materials & experimental systems

| n/a | Involved in the study | |
|---|---|---|
| ☐ | ☒ | Antibodies |
| ☐ | ☒ | Eukaryotic cell lines |
| ☒ | ☐ | Palaeontology and archaeology |
| ☐ | ☒ | Animals and other organisms |
| ☒ | ☐ | Human research participants |
| ☒ | ☐ | Clinical data |
| ☒ | ☐ | Dual use research of concern |

## Methods

| n/a | Involved in the study | |
|---|---|---|
| ☒ | ☐ | ChIP-seq |
| ☐ | ☒ | Flow cytometry |
| ☒ | ☐ | MRI-based neuroimaging |

## Antibodies

Antibodies used

Primary Antibodies (Used at 1:1000 dilution for WB, 1:500 dilution for Flow Cytometry)
Each antibody was researched prior to purchase and validated on a positive control in-house, making sure to confirm expected protein sizes for each target.
anti-CD44 (Biolegend, Cat#:338807): Manufacturer validated for Flow cytometry (+/- controls)
References: https://www.biolegend.com/en-gb/products/pe-anti-human-cd44-antibody-5745?GroupID=BLG6332
anti-MUCl (Sigma-Aldrich, 05-652): Manufacturer validated by immunohistochemistry
site lists several references: https://www.sigmaaldrich.com/US/en/product/mm/05652
anti-MUC4 (Thermofisher, 35-4900): Manufacturer validated by IHC, and site lists 18 references for assorted uses Western blot, IHC, Flow cytometry: https://www.thermofisher.com/antibody/product/MUC4-Antibody-clone-1G8-Monoclonal/35-4900
anti-MUC5AC (Abcam, ab198294): Manufacturer tested and recommended for WB, IHC-P, ICC/IF with positive and negative controls, site lists 6 references: https://www.abcam.com/mucin-5ac-antibody-epr16904-ab198294.html
anti-ROCKl (Proteintech, 21850-1-AP): Manufacturer tested for WB by ROCK1 siRNA inhibition, site lists 39 references for WB use : https://www.ptglab.com/products/ROCK1-Antibody-21850-1-AP.htm
anti-ACE2 antibody (Proteintech, 2115-1-AP): Manufacturer tested WB in various tissues, site lists 42 references for WB use https://www.ptglab.com/products/ACE2-Antibody-21115-1-AP.htm
Loading control: HRP-conjugated primary anti-B-actin (Santa Cruz Biotechnologies, sc-47778 HRP) at a 1:1000 dilution. Manufacturer validates with positive controls, site lists over 11,000 references (as this is a common loading control protein): https://www.scbt.com/p/beta-actin-antibody-c4
Secondary Antibodies: Secondary antibodies: anti-mouse lgG: LiCor, Cat#: 926-32210, anti-rabbit secondary antibody (LiCor, 926-32211)



April 2020

| | |
|---|---|
| Validation | Each antibody was compared to an isotype control and cellular lysates from both positive and negative controls by the vendors, all antibodies have well documented references, and in house validation included positive controls, and confirmation of expected protein sizes to confirm binding specificity. |

## Eukaryotic cell lines

Policy information about cell lines

| | |
|---|---|
| Cell line source(s) | ATCC: Calu-3 Catolog #: HTB-55, Vero-E6 Catolog #: CRL-1586, HEK293T Catalog #: CRL-1573/LONZA: Normal Human Bronchial Epithelial Cells (NHBE, Catalog #: CC-2541) |
| Authentication | Obtained from ATCC via the UC Berkeley Cell Culture Facility (Calu-3/Vero-E6), or Lonza (NHBE). Calu-3 cells were stained for adherens junction marker E-cadherin to confirm epithelial cell-like properties, also ATCC performed STR profiling. HEK293's were validated by STR profiling by ATCC. No VeroE6 or NHBE authentication was found on the vendors websites. |
| Mycoplasma contamination | Cells were certified mycoplasma free from the UC Berkeley Cell Culture Facility/Lonza upon receipt. |
| Commonly misidentified lines (See ICLAC register) | No commonly misidentified cell lines were used in this study. |

## Animals and other organisms

Policy information about studies involving animals; ARRIVE guidelines recommended for reporting animal research

| | |
|---|---|
| Laboratory animals | Our study involved mice from a C57BL/6 background of both genders conducted in the University of North Carolina Animal Facility in accordance with all state and federal regulations. All animal work was approved by Institutional Animal Care and Use Committee at University of North Carolina at Chapel Hill according to guidelines outlined by the Association for the Assessment and Accreditation of Laboratory Animal Care and the U.S. Department of Agriculture. All infection studies were performed in animal biosafety level 3 (BSL-3) facilities at University of North Carolina at Chapel Hill. 15-20 week old C57BL/6NTac mice, of both genders, were used for in vivo experiments. All mice were housed in individually ventilated microisolator cages in a facility maintained at the University of North Carolina at Chapel Hill, on a 12-h day/night cycle. Mice were fed a regular chow diet and given water ad libitum until the defined experimental endpoints. Researchers were not blinded during in vivo experiments. |
| Wild animals | This study did not involve wild animals. |
| Field-collected samples | This study did not involve samples collected from the field. |
| Ethics oversight | All animal work was approved by Institutional Animal Care and Use Committee at University of North Carolina at Chapel Hill according to guidelines outlined by the Association for the Assessment and Accreditation of Laboratory Animal Care and the U.S. Department of Agriculture. All infection studies were performed in animal biosafety level 3 (BSL-3) facilities at University of North Carolina at Chapel Hill. |

Note that full information on the approval of the study protocol must also be provided in the manuscript.

## Flow Cytometry

### Plots

Confirm that:

☒ The axis labels state the marker and fluorochrome used (e.g. CD4-FITC).

☒ The axis scales are clearly visible. Include numbers along axes only for bottom left plot of group (a 'group' is an analysis of identical markers).

☒ All plots are contour plots with outliers or pseudocolor plots.

☒ A numerical value for number of cells or percentage (with statistics) is provided.

### Methodology

| | |
|---|---|
| Sample preparation | Calu-3 cells were uplifted and treated with 5ug/mL StcE at 37C for an hour. Cells were then spun down, resuspended in block buffer for 10 minutes at room temperature (stain buffer, BD Pharmigen) supplemented with 2% FBS, and 0.1% BSA (Thermofisher)). Cells were then incubated in a 1:500 dilution of PE-conjugated anti-CD44 antibody (Biolegend, Cat#:338807) in stain buffer for 30 minutes at room temperature. After 3 washes with stain buffer cells were analyzed by flow cytometry. |
| Instrument | Attune NxT |
| Software | FlowJo |
| Cell population abundance | After gating for live and single cells, all populations had greater than 10,000 cells. Exact numbers are now reported in main text, figure legends. |

| Gating strategy | Cells were gated based on FSC/SSC to select for live cells. Then FSC-H/FSC-A to select for only single-cells, rather than aggregates. These live, single cells were then plotted for RL-1 signal to detect PE signal of the anti-CD44 antibody. |
|---|---|

☒ Tick this box to confirm that a figure exemplifying the gating strategy is provided in the Supplementary Information.
