# Supplementary material for: Genome-wide bidirectional CRISPR screens identify mucins as host factors modulating SARS-CoV-2 infection
Source: Nat Genet. 2022 Jul 25;54(8):1078–89. doi: 10.1038/s41588-022-01131-x (PMC9355872; doi:10.1038/s41588-022-01131-x)
Supplement: Supplementary file 4 — Unprocessed western blots and/or gels. [file 41588_2022_1131_MOESM4_ESM.pdf]

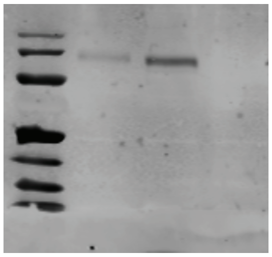

Extended Figure 3g
Probed anti-ACE2
Imaged on fluroescent channel

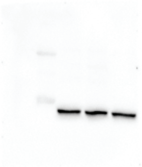

Extended Figure 3g
Probed anti-B-actin (loading control)
Imaged in chemiluminescent channel (HRP)
