# Supplementary material for: Genome-wide bidirectional CRISPR screens identify mucins as host factors modulating SARS-CoV-2 infection
Source: Nat Genet. 2022 Jul 25;54(8):1078–89. doi: 10.1038/s41588-022-01131-x (PMC9355872; doi:10.1038/s41588-022-01131-x)
Supplement: Supplementary file 6 — Unprocessed western blots and/or gels. [file 41588_2022_1131_MOESM6_ESM.pdf]

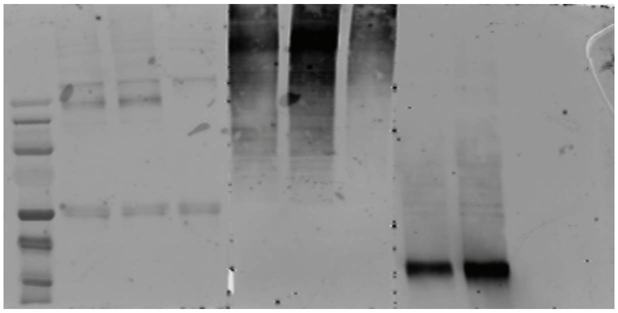

Extended Figure 6b
Gel ran together
Lane 1: Ladder
Lanes 2-4: MUC4
Lanes 5-7: MUC5AC (Extended Figure 9b)
Lanes 8-10: MUC1
Membrane was cut, antibody staining done individually
Imaged together, aligned on fluorescnet channel

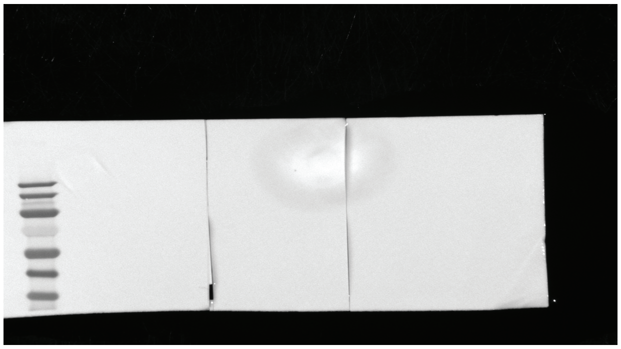

Extended Figure 6b
Lane 1: Ladder
Lanes 2-4: MUC4
Lanes 5-7: MUC5AC Extended (Figure 9b)
Lanes 8-10: MUC1
Brightfield accompanying anti-B-Actin Stain
(same orientation, can be overlayed)

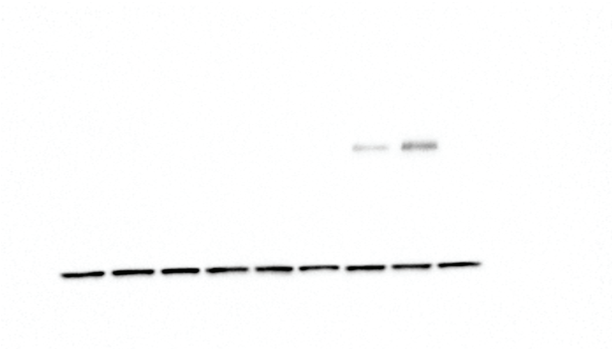

Extended Figure 6b
Probed anti-B-actin (loading control)
Imaged in chemiluminescent channel (HRP)

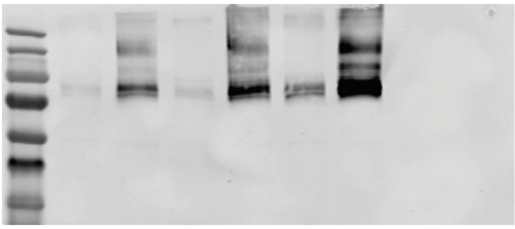

Extended Figure 6c
Probed anti-CD44
Imaged on fluorescent channel

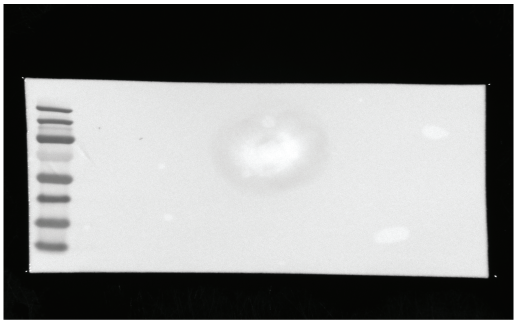

Extedned Figure 6c
Probed anti-B-actin (loading control)
Imaged in chemiluminescent channel (HRP)

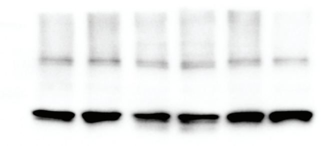

Extended Figure 6c
Brightfield shot accompanying anti-B-Actin Stain
(same orientation, can be overlayed)
