# Supplementary material for: Genome-wide bidirectional CRISPR screens identify mucins as host factors modulating SARS-CoV-2 infection
Source: Nat Genet. 2022 Jul 25;54(8):1078–89. doi: 10.1038/s41588-022-01131-x (PMC9355872; doi:10.1038/s41588-022-01131-x)
Supplement: Supplementary file 7 — Unprocessed western blots and/or gels. [file 41588_2022_1131_MOESM7_ESM.pdf]

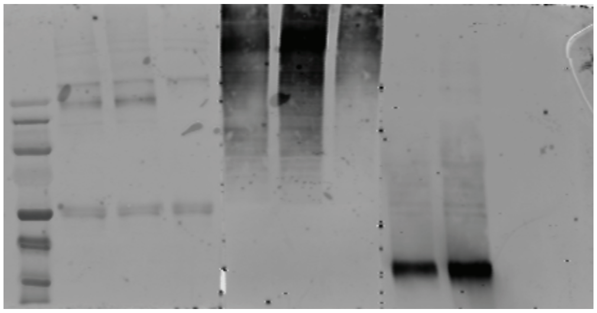

Extended Figure 9b
Gel ran together
Lane 1: Ladder
2-4: MUC4 (Extended Figure 6b)
5-7: MUC5AC
8-10: MUC1 (Extended Figure 6b)
Membrane was cut, antibody staining done individually
Imaged together, aligned on fluorescent channel

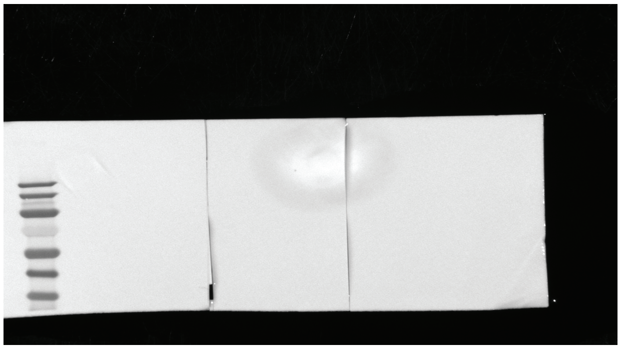

Extended Figure 9b
Brightfield accompanying anti-B-Actin Stain
(same orientation, can be overlayed)

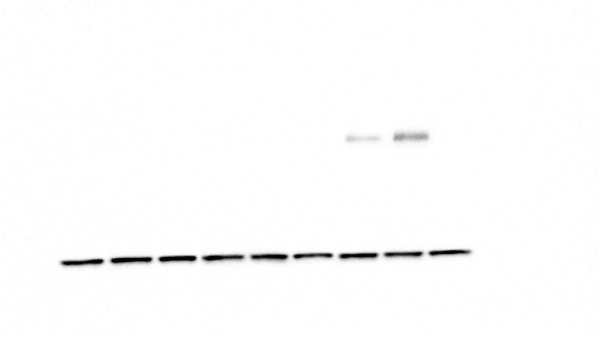

Extended Figure 9b
Probed anti-B-actin (loading control)
Imaged in chemiluminescent channel (HRP)
